# Spatio-temporal dynamics of phytoplankton community in a well-mixed temperate estuary (Sado Estuary, Portugal)

**DOI:** 10.1038/s41598-022-20792-6

**Published:** 2022-09-30

**Authors:** M. Santos, A. Amorim, V. Brotas, J. P. C. Cruz, C. Palma, C. Borges, L. R. Favareto, V. Veloso, M. L. Dâmaso-Rodrigues, P. Chainho, P. M. Félix, A. C. Brito

**Affiliations:** 1 MARE – Marine and Environmental Sciences Centre/ARNET - Aquatic Research Network, Faculdade de Ciências, Universidade de Lisboa, 1749-016 Lisboa, Portugal; 2 Departamento de Biologia Vegetal, Faculdade de Ciências, Universidade de Lisboa, 1749-016 Lisboa, Portugal; 3 Instituto Hidrográfico, Rua das Trinas 49, 1249-093 Lisboa, Portugal; 4 Departamento de Biologia Animal, Faculdade de Ciências, Universidade de Lisboa, 1749-016 Lisboa, Portugal; 5 Escola Superior de Tecnologia de Setúbal - CINEA, Instituto Politécnico de Setúbal, 2914-508 Setúbal, Portugal

**Keywords:** Ecology, Ecology, Environmental sciences, Ocean sciences

## Abstract

Estuaries are highly productive ecosystems, which are strongly affected by several anthropogenic pressures. Phytoplankton is a key element for assessing the ecological quality status in these transitional waters. Moreover, understanding physico-chemical and biological drivers is crucial to disentangle their effect on the structure of phytoplankton community. The present work aims to study the effect of the main physico-chemical drivers on the phytoplankton community structure and dynamics in a temperate well-mixed estuary (Sado Estuary). Four sampling stations were analyzed monthly in three regions of the estuary, from 2018 to 2019. Surface water samples were collected to analyze the phytoplankton community and several concomitant physico-chemical parameters. Temperature, turbidity, salinity, and nutrients availability were the drivers that best explained the spatio-temporal patterns observed in the phytoplankton community. The upper estuary was characterized by higher phytoplankton cell abundances and biomass. Three phytoplankton groups stood out in the characterization of the estuarine assemblages: diatoms, cryptophytes, and dinoflagellates. Diatoms were the dominant group most of the year, being dominated by small cell species (single and chain-forming) upstream, and by larger chain-forming species downstream. Cryptophytes had a high contribution to the community in the inner regions of the estuary, while dinoflagellates contributed more for the community composition downstream, where high abundances of harmful algal species were sporadically found. Previous studies on the phytoplankton community dynamics in this estuary are limited to the 1990s. Thus, the present study provides insight into changes in the dominant phytoplankton groups of the Sado Estuary in the last 25 years, namely an increase in cryptophytes over diatoms in the inner estuarine regions, and an increase in dinoflagellates near the estuary mouth.

## Introduction

Estuaries are among the most productive ecosystems in the world. Estuarine ecosystems play an important role in providing food and resting areas for higher trophic levels, such as marine mammals, fishes and migratory bird species, and nursery habitats for several species within oyster reefs, seagrass beds and wetlands^1^. They also provide benefits to society through services provided by the ecosystem, such as climate regulation, food production (e.g., fisheries and shellfish aquaculture) and recreation^1^.

However, estuaries are also some of the most stressed ecosystems, suffering several anthropogenic impacts and fluctuations in their environmental conditions. Phytoplankton communities are highly sensitive to changes in the environment, being considered good biological indicators of water quality and of ecosystem health under different environmental pressures, such as eutrophication^2,3^. In several estuaries, eutrophication has been observed to generate high concentrations of opportunistic phytoplankton species, some of them toxic or harmful, which may contribute to oxygen depletion and harmful impacts on fish and shellfish resources and, subsequently, on human health^4,5^.

Phytoplankton biomass and communities vary greatly in space and time under the influence of different physico-chemical, hydrological, and biological drivers. Drivers such as nutrients (which may be originated from different sources: upwelling, river discharge, or land runoff), water temperature, salinity, light availability, turbidity, and grazing are responsible for shaping phytoplankton communities, through bottom-up or top-down effects^6^.

In the European Union, the Water Framework Directive (WFD)^7^ aims to achieve a Good Ecological Status (GES) for all European surface water bodies (WBs). In this directive, several phytoplankton indicators are used to evaluate the ecological quality status (EQS) of both transitional and coastal waters, namely: i) phytoplankton biomass; ii) taxonomic composition and cell concentration; and iii) frequency and intensity of blooms^8^. However, in Portugal, only the method focused on phytoplankton biomass has been developed^9,10^, and according to this method the Sado Estuary is currently classified as High ecological condition.

The Sado Estuary is the second-largest estuary in Portugal (with an area of 180 km^2^) and one of the most important wetlands in the national territory, integrating an important natural reserve since 1980. It is one of the main areas for aquaculture in Portuguese brackish waters, playing an important role in the local and national economy^11^. The Sado Estuary is under high levels of anthropogenic pressure due to an intense human occupation, with the city of Setúbal (one of the main Portuguese commercial and fishing ports) located in the northern margin. In this estuary there are a variety of activities ranging from industrial (e.g., paper mills, metal processing, shipyards, thermo-electrical production, plastic components and packing, car repair works), agriculture (e.g., rice production), fisheries and aquaculture, and tourism activities^12^.

With low freshwater flow rate draining to the estuary and a clear seasonal variability (from values lower than 1 m^3^ s^−1^ in summer to frequent winter average values of 60 m^3^ s^−1^)^13–15^, Sado Estuary is characterized as being a low-inflow system. Given that the nearby coastal region is influenced by seasonal upwelling, typical features of these systems include an outer region characterized by thermal effects (i.e., warm estuarine waters *versus* cold upwelled ocean waters), while hypersaline conditions can be observed in the inner region^16^. Since the 1960s, a number of studies published in grey literature, such as reports and theses, have focused on the phytoplankton communities in the Sado Estuary. However, those studies did not cover spatially the entire estuarine system and occurred in small temporal scales or few seasons^17–21^. Only a doctoral thesis published in 2003 presents the first complete spatial assessment of the phytoplankton communities in the Sado Estuary, covering the period between April 1992 and May 1993^22^. This work was a great contribution to the knowledge of the spatial and seasonal variability and dynamics of the phytoplankton in the estuary^22^. Those studies previously conducted in the Sado Estuary, particularly the latter, showed that higher chlorophyll *a* concentrations characterized the upper estuary, compared to the lower estuarine regions, with diatoms being the dominant group in the entire system^9,23–26^.

The present work will contribute to the knowledge with a recent characterization of phytoplankton structure and dynamics in the Sado Estuary, where changes in the phytoplankton community for the last two decades will be investigated. The main aims of this study were: (i) to evaluate the spatio-temporal variation of the phytoplankton community in the Sado Estuary; and (ii) to understand how physico-chemical drivers influence the phytoplankton dynamics. By allowing a better understanding of natural capital, this study represents an important contribution to the sustainability of the Sado Estuary.

## Methods

### Study area and sampling strategy

**Figure 1:**
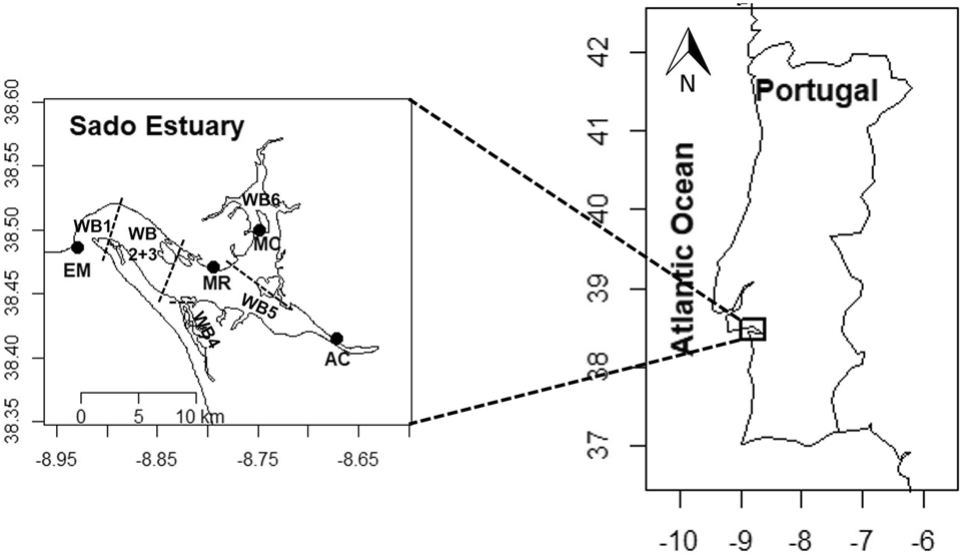
Map of the study area, Sado Estuary, with the location (black dots) and identification of each study site: AC—Alcácer channel, MC—Marateca channel, MR—middle region, and EM—estuary mouth. The maps were produced using the ‘maps’ package (3.4.0) from R (4.2.028). The water bodies (WB) currently used in the context of the WFD^72^ are also shown.

The present study was conducted in the Sado Estuary, located in the southwestern Portuguese coast (Fig. 1). The Sado Estuary is a well-mixed estuary, with a low average depth, strong tidal currents, little to no stratification^15^, and a mean residence time of 21 days^14^. The circulation in this estuary occurs through two navigation channels (North and South Channels), with the South channel being the main route for water exchange^15^. The oceanic water influences the entire Sado Estuary, with high salinity values observed throughout the whole estuary, reaching values near 35 during summer in the innermost regions^26^, and with upwelling influencing phytoplankton biomass especially near the estuary mouth^27,28^.

Under the WFD, the Sado Estuary is divided in six water bodies (WB) (Fig. 1). This division considers the differentiation in terms of morphology and pressures. In this study, four sampling stations were analyzed in three different water bodies (WB 1, 5 and 6) (Fig. 1): (i) one station located upstream near the Alcácer channel (AC), WB5; (ii) one located northward, in the Marateca channel (MC), WB6; (iii) one in the middle estuarine region (MR), WB5; and (iv) one in the downstream area, near the estuary mouth (EM), WB1. Stations EM and MR in this study correspond, respectively, to stations A and D in a previous study performed in this estuary during the same period, focused on the effect of the tidal variability in the water quality parameters^27^.

The Alcácer channel has an average depth of 5 m and is where about 80% of the total freshwater enters the estuary, while the Marateca channel is responsible only for about 10% of the total inflow^14^. The remaining freshwater inflow comes from small streams that also discharge in this estuary. The middle estuary is a wide embayment with an average depth of 10 m^14^, and downstream, the estuary mouth connects with the Atlantic Ocean through a deep narrow channel of about 38 m depth^15^.

In this work, in order to analyze several physico-chemical and biological elements, water samples were collected monthly, at surface, between March 2018 and November 2019, covering the high tide condition. The geographical coordinates and respective depths of the sampling stations can be found in Table S1 of the Supplementary Materials. Samples were occasionally not collected due to weather conditions and lost due to methodological constraints, which resulted in missing data in different physico-chemical and/or biological variables.

### Physico-chemical data

Several physico-chemical parameters were measured in situ or in the laboratory by collecting surface (< 1 m depth) water samples using 8 L Niskin bottles: water temperature, salinity, pH, dissolved oxygen (DO), turbidity, coloured dissolved organic matter, and dissolved nutrients—nitrite (NO_2_^−^), nitrate (NO_3_^−^), ammonium (NH_4_^+^), phosphate (PO_4_^3−^) and silicate (Si(OH)_4_). The dissolved inorganic nitrogen (DIN) is presented as the sum of NO_2_^−^, NO_3_^−^ and NH_4_^+^. Water temperature (ºC) and DO were measured in situ using two multiparameter sondes (Hydrolab DS4a and DS5X, from OTT Hydromet), while salinity, pH and nutrient concentrations (µmol L^−1^) were measured in the laboratory using, respectively, a high precision Guildline Autosal8400B salinometer, a calibrated Metrohm benchtop 744 pH meter, and a Skalar SANplus autoanalyzer specially engineered for the analysis of saline waters by UV/Vis spectrometry. Further details on the methodologies used are described in Nascimento et al.^27^. Turbidity was also measured in the laboratory by the nephelometric method using a portable infrared Lovibond TB 210IR turbidity meter, following the methodology described in Sent et al.^26^. Coloured dissolved organic matter (CDOM) was obtained through spectrophotometry (UV-2600 Shimadzu), also according to the methodology described in Sent et al.^26^ and was calculated at 443 nm wavelength. Daily precipitation data were obtained from the SNIRH—Sistema Nacional de Informação de Recursos Hídricos—database (https://snirh.apambiente.pt/), from the meteorological station at “Montevil”. Since there are a lot of missing data in Sado River runoff estimates for the years 2018/2019, available from the SNIRH database, this hydrological parameter was not considered in this study.

### Phytoplankton sampling and laboratory procedures

To analyze the chlorophyll *a* (Chl-*a*) concentration, ca. 1 L of water samples collected at surface were filtered, extracted by a mixture of Acetone:Water 9:1 (v:v) and analyzed in the laboratory using a ThermoFisher Scientific Evolution 201 spectrophotometer, according to Nascimento et al.^27^. The absorbance was read using a spectrophotometer (UV–VIS Unicam UV2-100 and ThermoScientific Evolution201) at 664 nm to determine Chl-*a* content. Absorbance at 750 nm was read to correct the influence of turbidity on the sample. Chlorophyll *a* concentrations were obtained using the expressions recommended by UNESCO, the Jeffrey and Humphrey^30^ equations.

To analyze the phytoplankton community, water samples were collected at surface (125 mL) and field fixed with acid Lugol’s solution^30^. Phytoplankton identification and quantification (cells L^−1^) was carried out by settling 10–50 mL of water, following the Utermöhl method^31^. The samples were analyzed with an inverted microscope equipped with phase contrast and bright field illumination (Zeiss Axiovert 200 and Zeiss AxioVert A1) at magnifications of 200x and 400x. More than 200 cells were identified and counted at the lower magnification to achieve a representative sample. The smallest cells (< 15 µm) were identified by counting the two diameters of the sedimentation chamber at the highest magnification. Phytoplankton species or genera for which there were doubts in the identification were grouped and reported as taxonomic entities (e.g., *Dactyliosolen blavyanus/Guinardia flaccida, Heterocapsa* spp.*/Azadinium* spp, see Table S2 in the Supplementary Material). Many specimens were not resolved at species level, i.e., they were grouped at the genus level, with no size-class distinction, or even grouped in wide functional groups such as athecate or small dinoflagellates. All taxonomic entities were posteriorly merged in higher taxonomic groups: Baccilariophyceae, Dinophyceae, Cryptophyceae, Euglenophyceae, Prasinophyceae, Chlorophyceae, Crysophyceae, Cyanophyceae, Prymnesiophyceae, and other small flagellates. Coccolithophores were not considered in the analysis since the acid version of the fixative was used. Phytoplankton identification was mostly based on the descriptions made by Dodge^32^, Hoppenrath et al^33^, Peragallo and Peragallo^34^, Schiller^35^, and Thomas^36^. An effort was made to use the currently accepted taxonomic names following the AlgaeBase database^37^ (https://www.algaebase.org/).

### Data processing and statistical analysis

Multivariate analyses were performed using PRIMER-E (version 6.1.13) with PERMANOVA (version 1.0.3) add-on software^38,39^ to analyze the spatio-temporal phytoplankton community structure and the underlying physico-chemical drivers. The taxonomic entities that occurred in less than 5% of the total samples (n = 79) were excluded from the analysis, therefore only 114 of the 211 identified taxonomic entities were considered in the analysis. To reduce the disproportionate influence of highly abundant taxa, phytoplankton abundances were log (x + 1) transformed. The Shannon–Wiener diversity index (*H*′ = − Σ*Pi* log *Pi,* where *Pi* = *ni*/*N*) was calculated using the routine DIVERSE. The evenness of the numbers of phytoplankton taxonomic entities in each sample was also calculated using Pielou's index (*J*′ = *H*′/log*S*, where log*S* = *H*′_max_).

A permutational analysis of variance (PERMANOVA) was performed with 999 permutations for three fixed factors (year—2 levels, study site—4 levels and meteorological season—4 levels), based on a Bray–Curtis resemblance matrix, using the phytoplankton abundances data. When the number of unique permutations is small (< 100), Monte Carlo tests were performed. These analyses were carried out at a level of α = 0.05. Additionally, a similarity percentages routine (SIMPER) was conducted to identify the taxonomic entities that contributed most to differences in the phytoplankton assemblage structure between the sampling years.

A Principal Coordinates Analysis (PCO) was used to visualize the multivariate patterns of the global phytoplankton composition and to explore the relationship between the environmental variables and phytoplankton composition. All physico-chemical variables were previously tested for collinearity and only those with correlations smaller than 0.7 were considered, namely water temperature, pH, turbidity, dissolved inorganic nitrogen, phosphate, silicate, and the 7-day average of precipitation that occurred one week before sampling. Spearman correlations of the physico-chemical drivers and the taxonomic entities with the PCO axes were performed, allowing to highlight the environmental drivers and species most correlated with the spatial and temporal patterns observed on phytoplankton assemblages.

## Results

### Physico-chemical characterization of the Sado Estuary

**Figure 2:**
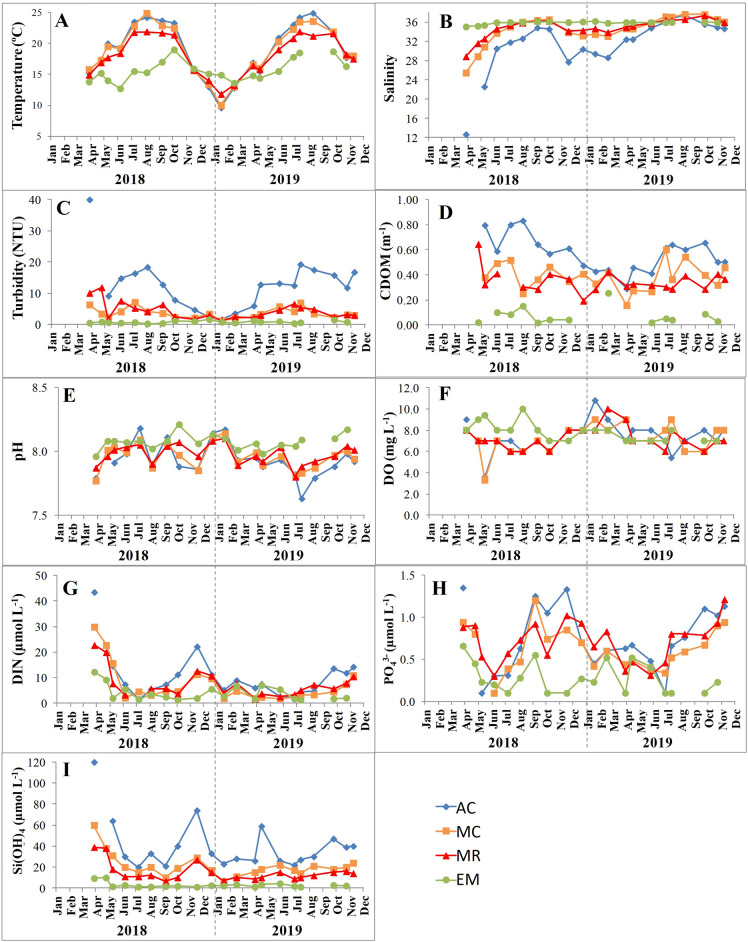
Discrete time series of physico-chemical variables obtained in the Sado Estuary during sampling surveys. (**A**)—Water temperature (°C); (**B**)—Salinity; (**C**)—Turbidity (NTU); (**D**)—Coloured dissolved organic matter at 443 nm (CDOM, m^−1^); (**E**)—pH; (**F**)—Dissolved oxygen (DO, mg L^−1^); (**G**)—Dissolved inorganic nitrogen (DIN, µmol L^−1^); (**H**)—Phosphate (PO_4_^3−^, µmol L^−1^); and (**I**) –Silicate (Si(OH)_4_, µmol L^−1^).

The seasonal cycle of water temperature in the Sado Estuary in 2018 and 2019, showed the expected pattern, with maxima temperature observed in summer and minima in winter (Fig. 2A). During summer, warmer temperatures were found in the inner regions of the estuary (AC and MC) and lower temperatures near the mouth of the estuary (EM). During winter, there was an inversion of the pattern, with the coolest waters recorded inside the estuary (Fig. 2A). Near the estuary mouth (EM), salinities recorded were always between 35 and 36 (Fig. 2B). In the inner stations, higher salinities (> 30) were found during summer/early-autumn of 2018 and late-spring/summer of 2019. Maxima salinities (> 36) were recorded in the summer of 2019 (Fig. 2B). The lowest salinities were always found in the upper region (AC), reaching a minimum of 12 in March 2018 (Fig. 2B).

The water turbidity was substantially higher in AC, reaching values above 10 NTU in the summer of 2018 and since spring of 2019, with a maximum of 40 NTU recorded in March 2018 (Fig. 2C). The turbidity values and seasonal pattern for stations MC and MR were similar, with a maximum of 10 NTU recorded in MR during spring of 2018 (Fig. 2C). Lower turbidity was observed during winter in stations AC, MC, and MR (Fig. 2C). Water turbidity was always lower than 1.5 NTU at EM (Fig. 2C). The CDOM was higher in the upper region and lower in the downstream area (Fig. 2D). At AC, a CDOM value < 0.45 m^−1^ was recorded between winter 2018 and spring 2019. In the remaining period, values above 0.60 m^−1^ were observed only in May, July, and August of 2018. At MC and MR, CDOM varied between 0.15 m^−1^ and 0.65 m^−1^ (Fig. 2D). In the downmost station (EM), CDOM was always below 0.15 m^−1^, except for February 2019, which reached 0.25 m^−1^ (Fig. 2D).

The pH observed in both studied years varied mainly from 7.8 to 8.2, except for July of 2019 at AC, where the minimum value was recorded (7.6) (Fig. 2E). Only the station located near the estuary mouth (EM) registered pH values always above 8.0 (Fig. 2E). The dissolved oxygen (DO) concentration ranged between 6 and 10 mg L^−1^ in all stations (Fig. 2F). From April to October of 2018, higher DO concentrations were observed near the estuary mouth, when compared with the other stations. The minimum DO concentration was observed in May of 2018 (3.3 mg L^−1^), simultaneously at AC and MC (Fig. 2F).

An increase in nutrient concentrations (DIN, phosphate, and silicate) was found from downstream towards upstream stations, with higher concentrations in 2018 than in 2019 (Fig. 2G,H,I). DIN reached maxima above 15 µmol L^−1^ in spring (AC, MC, and MR) and autumn (AC) of 2018. In 2019, lower DIN concentrations were observed in the entire estuary, reaching maxima values during autumn in AC (> 10 µmol L^−1^) (Fig. 2G). Phosphate concentrations were below 1.5 µmol L^−1^ in the entire estuary, with higher values in the inner stations, and lower (< 0.6 µmol L^−1^) near the estuary mouth (Fig. 2H). In the inner stations, higher concentrations were recorded during spring and autumn of 2018, and since mid-summer of 2019 (Fig. 2H). Silicate concentrations had two peaks each year in AC (in spring and autumn), being higher in 2018 than in 2019 (Fig. 2I). These two peaks could also be found during 2018 in MC and MR. Near the estuary mouth, silicate concentrations were always low (< 10 µmol L^−1^) (Fig. 2I).

**Figure 3:**
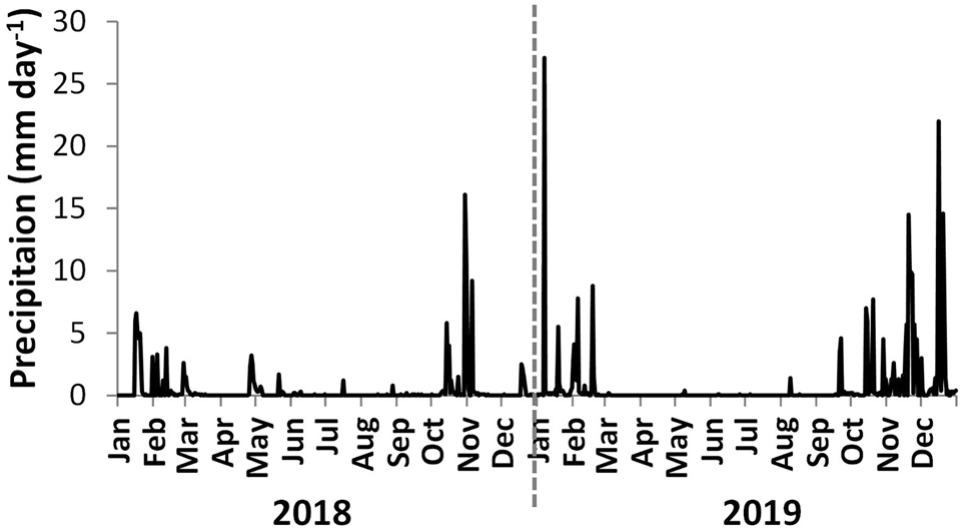
Daily time series of precipitation measured in the Sado Estuary region in the meteorological station of “Montevil” (mm day^−1^, https://snirh.apambiente.pt/).

The daily precipitation measured in the Sado Estuary showed higher values in 2019 than in 2018 (Fig. 3). Precipitations above 5 mm day^−1^ were recorded in winter and autumn of both years, with maxima (above 15 mm day^−1^) observed in late-October of 2018, January, and December of 2019 (Fig. 3).

**Figure 4:**
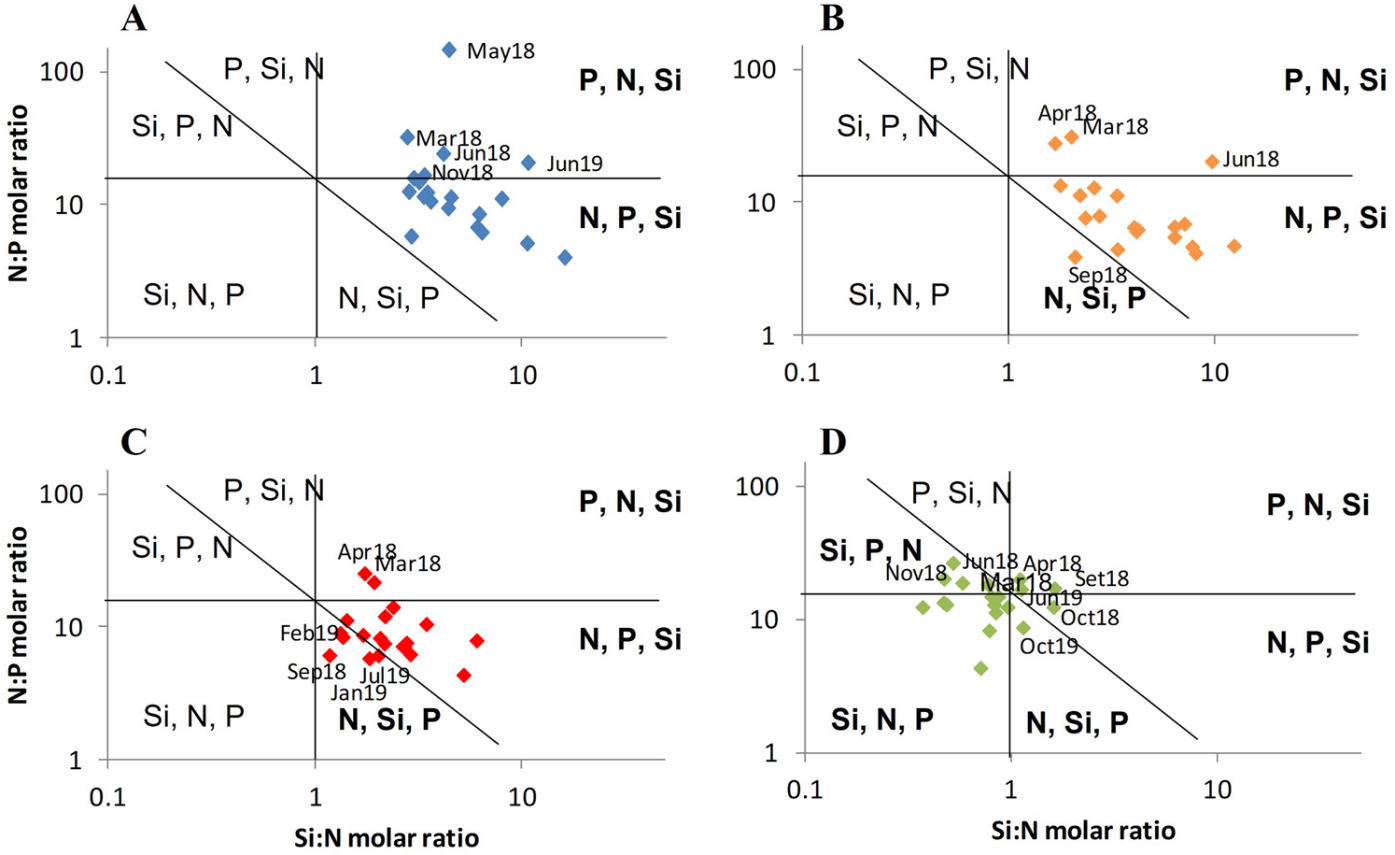
Si:N:P molar ratios in the Sado Estuary during sampling: (**A**)—AC, (**B**)—MC, (**C**)—MR and (**D**)—EM. Horizontal lines—N:P ratio^40^; vertical lines—Si:N ratio^41^; diagonal lines—aggregate ratio (Si:N:P = 16:16:1). These figures define six different areas characterized by the potentially limiting nutrients in order of priority^51^. The areas where this study samples are included are highlighted in bold.

Figure 4 shows the representation of molar quotients between the concentrations of potentially limiting nutrients for phytoplankton growth for each station, which are delimited by the Si:N = 1, N:P = 16 and Si:P = 16 lines^40,41^. Inner stations of the estuary (AC, MC and MR) had a higher availability of silicate, as Si:N and Si:P were mostly recorded above the ratio (Fig. 4A,B,C), whereas, near the estuary mouth (EM), there was a high number of samples with Si:N lower than 1, and Si:P lower than 16 (Fig. 4D). The N:P ratios above 16 were recorded especially during spring and summer of 2018, namely in March, May, and June at AC (Fig. 4A), in March, April and June at MC (Fig. 4B), in March and April at MR (Fig. 4C) and in March, April and June at EM (Fig. 4D).

### Intra- and inter-annual variation of phytoplankton

**Figure 5:**
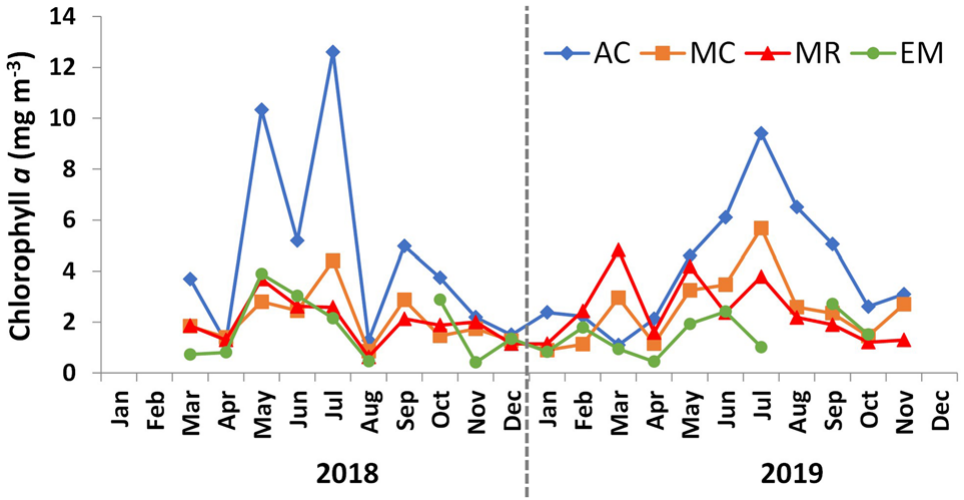
Chlorophyll *a* concentration (mg m^−3^) obtained in the Sado Estuary during sampling: AC—Alcácer channel, MC—Marateca channel, MR—middle region, EM—estuary mouth. No data available at EM for September 2018 and August 2019.

Phytoplankton biomass (Chl-*a*) was consistently higher at AC in both years, reaching concentrations above 8 mg m^−3^ in May and July 2018 and July 2019 (Fig. 5). In the other three studied sites, Chl-*a* was always below 6 mg m^−3^. In these stations, during 2018, Chl-*a* maxima were observed in May at EM and MR, and in July at MC. During 2019, both AC and MC peaked in July, whereas in EM it was no clear peak (Fig. 5). There were three Chl-*a* peaks at MR during 2019, which were recorded in March, May, and July (Fig. 5).

**Figure 6:**
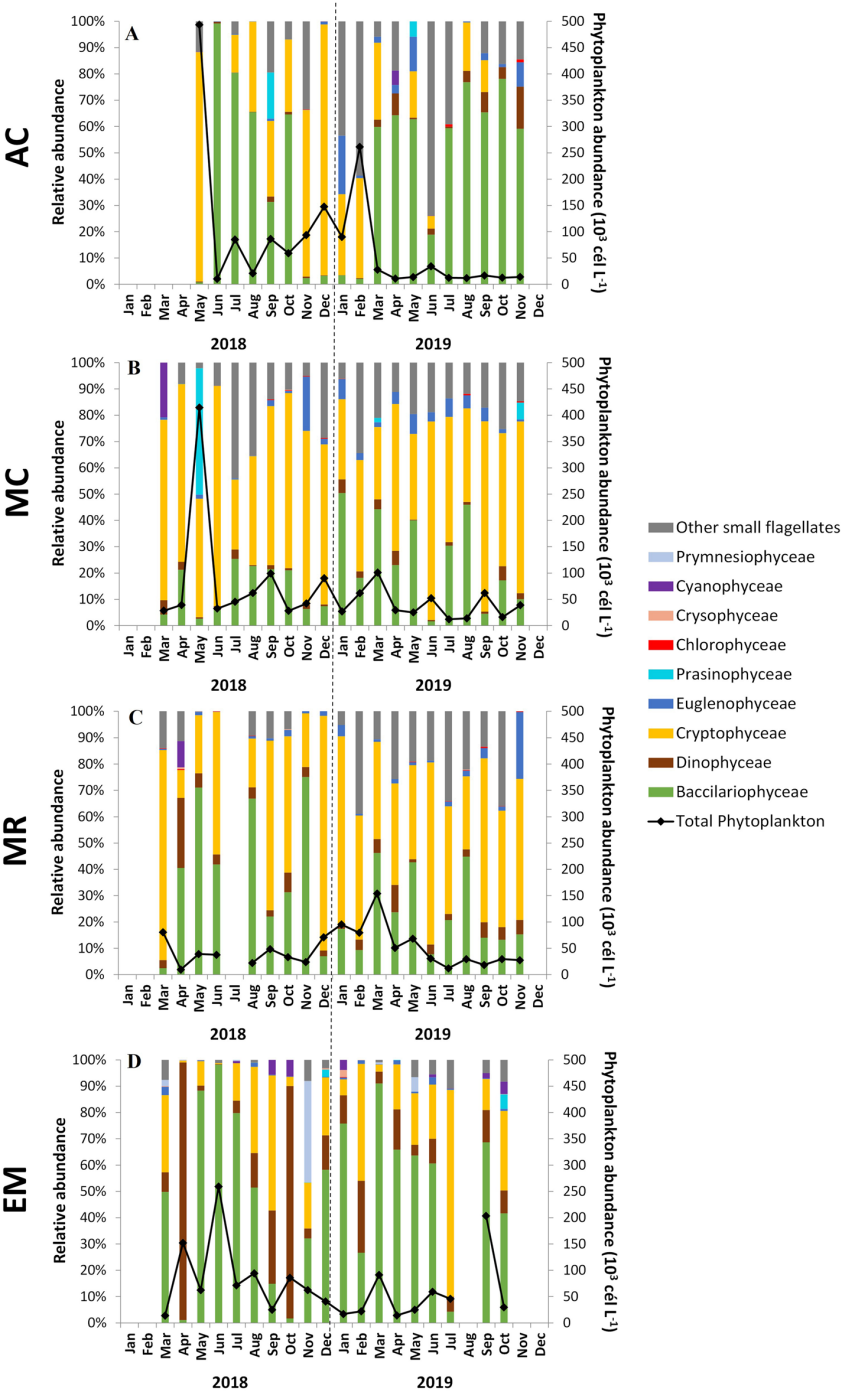
Relative abundance of different phytoplankton groups (colored bars) and phytoplankton cell abundance (× 10^3^ cell L^−1^) (dots with connecting line) obtained for each sampling month in the different study sites: (**A**)—AC, (**B**)—MC, (**C**)—MR and (**D**)—EM. No data available in July 2018 at MR and in August 2019 at EM. See the legend figure for the phytoplankton groups color.

Phytoplankton cell abundance for each studied site, as well as relative abundances of the phytoplankton groups identified are presented in Fig. 6. During 2018, AC and MC reached phytoplankton maximal abundances in May (above 400 × 10^3^ cell L^−1^) (Fig. 6A,B), the station located downstream (EM) reached the maximum abundance in June (ca. 250 × 10^3^ cell L^−1^) (Fig. 6D), while MR had low abundances all year round, with a maximum recorded in March (80 × 10^3^ cell L^−1^) (Fig. 6C). In 2019, a peak was recorded in February at AC (ca. 250 × 10^3^ cell L^−1^), but in the remaining year phytoplankton abundance was always below 50 × 10^3^ cell L^−1^ (Fig. 6A). In MC and MR, the maxima were recorded in March (respectively 100 × 10^3^ cell L^−1^ and 150 × 10^3^ cell L^−1^) (Fig. 6B,C). A high cell concentration was also recorded in March in the downmost station, with the peak being observed in September (200 × 10^3^ cell L^−1^) (Fig. 6D).

Results of the phytoplankton community at the class level evidenced the differences between the estuary mouth and the stations located inside the estuary, with cryptophytes being a highly dominant group recorded inside the estuary (AC, MC, and MR) (Fig. 6A,B,C). Several inter-annual differences could be observed. At AC, cryptophytes (Cryptophyceae) were more abundant during 2018, while in 2019 diatoms (Bacillariophyceae) dominated (Fig. 6A). A change in the community during late-autumn/winter could also be observed, with a reduction of diatoms and an increase in cryptophytes (Fig. 6A). At this station, it was also possible to observe a higher contribution of dinoflagellates (Dinophyceae) during 2019 than in 2018 (Fig. 6A). At MC, cryptophytes dominated during the studied period, and diatoms constituted the second most abundant group in most months, presenting higher concentrations in 2019 than in 2018 (Fig. 6B). The dominant phytoplankton groups at MR were similar with the ones observed at MC. The main difference was a higher contribution of diatoms in 2018 at MR, than in MC (Fig. 6C). Near the estuary mouth (EM), diatoms were, in general, the dominant group (Fig. 6D). The main exceptions occurred in April and October of 2018 when dinoflagellates dominated, and in July of 2019 when cryptophytes dominated (Fig. 6D). In the above-mentioned situations of dinoflagellate dominance recorded at EM in 2018, the phytoplankton assemblage was mostly composed by *Prorocentrum cordatum* in April (ca. 150 × 10^3^ cell L^−1^) and dominated by *Gymnodinium* spp. and *Gymnodinium catenatum* in October (65 × 10^3^ cell L^−1^) (Table S2).

For the other identified groups, in general, Euglenophyceae were more abundant inside the estuary, and had higher abundances in 2019 (Fig. 6A,B,C). Cyanophyceae occurred only sporadically, reaching higher abundances during spring inside the estuary (Fig. 6A,B), and during autumn near the estuary mouth (Fig. 6C). Other groups also formed sporadic blooms, as Prasinophyceae in May 2018 at MC and Prymnesiophyceae (i.e., *Phaeocystis* spp.) in November 2018 at EM (Fig. 6B).

The Chl-*a* concentrations (maximum and average) obtained during this study, for the different regions of the Sado Estuary, were smaller than those obtained for other periods and similar locations (Table 1). Higher differences were observed in the inner regions of the estuary (e.g., maximum of 12.6 mg m^−3^ obtained in 2018–2020, compared with 25 mg m^−3^ obtained during the 1990s in the Alcácer channel), than near the estuary mouth (e.g., maximum of 3.9 mg m^−3^ obtained in 2018–2020 and 5 mg m^−3^ obtained for the other periods). Although using different metrics (Chl-*a* maxima in this study and Chl-*a* 90th percentile for the WFD), the Chl-*a* maxima here obtained in the different regions of the estuary agree with the ‘High’ classification (Table 1).

**Table 1:** Comparison of maximum (Max) and average (Avg) of Chl-*a* concentrations (mg m^−3^) and phytoplankton abundances (cell L^−1^) obtained during different periods in the Sado Estuary, in the three regions here studied.

Sado Estuary, Portugal							
Year	Region	Location	Chl-a (mg m^−3^)		Abundance (cell L^−1^)		Reference
			Max	Avg	Max	Avg	
2018–2019	Lower	Estuary mouth (EM)	3.9	1.7	260 × 10^3^	70 × 10^3^	This study
	Mid	Marateca channel (MC)	5.7	2.8	415 × 10^3^	60 × 10^3^	
	Upper	Alcácer channel (AC)	12.6	5.2	490 × 10^3^	80 × 10^3^	
1992–1993	Lower	Estuary mouth (E1)	5	3.5^(a)^	450 × 10^3^	200 × 10^3^	Coutinho^22^
	Mid	Marateca channel (E5/E6)	15	5.6^(a)^	2280/3300 × 10^3^	560–800 × 10^3^	
	Upper	Alcácer channel (E8)	25	14.7^(a)^	5300 × 10^3^	970 × 10^3^	
1967–1968	Lower	Estuary mouth	–	–	< 250 × 10^3^	< 70 × 10^3^	Sampayo^18^
1967	Lower	Estuary mouth	5.5	2.3	**–**	**–**	Silva et al.^17^

**Water quality ecological assessment**							
**Country**	**Region**	**Location**	**Chl-a P90 (mg m**^−**3**^**)**		**Phytoplankton bloom threshold (cell L**^−**1**^**)**	**Reference**	
			**H/G**	**G/M**			
Portugal	Coastal water	Portugal upwelling coast	4.5	8.2	1000 × 10^3^	Brito et al.^9,10^	
	Transitional water	Sado Estuary (salinity 5–25)	12	18	5000 × 10^3^	Brito et al.^9^	
		Sado Estuary (salinity > 25)	10	15	2500 × 10^3^		

In the lower section are shown indicative values for water quality ecological assessment. The Chl-*a* 90th percentiles (P90) (mg m^−3^) are shown for High/Good (H/G) and Good/Moderate (G/M) boundaries. The thresholds for the determination of the occurrence of a phytoplankton bloom (cell L^−1^) are also shown.

^(a)^This value indicates the Chl-*a* average for the entire referred region, not only for the indicated station.

Phytoplankton abundances obtained in the lower region showed similar results with those observations made for the 1960s (around 250 × 10^3^ cell L^−1^) but were lower than the obtained in the 1990s (450 × 10^3^ cell L^−1^) (Table 1). In the mid and upper estuarine regions, the phytoplankton abundances obtained in this study were much lower than the obtained during the 1990s (e.g., 490 × 10^3^ cell L^−1^ recorded in the Alcácer channel in 2018/19, while 5300 × 10^3^ cell L^−1^ were recorded in the same site in 1992/93). Phytoplankton maximal abundance recorded in this study were always below the thresholds established previously for the determination of the occurrence of a phytoplankton bloom in the Sado Estuary, as well as for the Portuguese upwelling coast (Table 1).

### Spatial and temporal patterns of phytoplankton assemblages

A total of 10 phytoplankton groups were identified in the Sado Estuary during this study, with an increasing number of taxonomic entities identified from upstream (87 at AC) to downstream stations (179 at EM, Table S2). The phytoplankton groups with a higher number of taxa identified were the Bacillariophyceae and the Dinophyceae, with nearly 50 more taxa of dinoflagellates observed near the estuary mouth when compared to the stations inside the estuary (Table S2).

The year 2018 was characterized by having a higher number of taxonomic entities identified in all stations, reaching the maximum at EM (Table 2). Except for MR, 2018 was also characterized by higher phytoplankton abundance, with the maximum recorded at AC (Table 2). Table 2 also shows the phytoplankton diversity (H’) and evenness (*J*) indices obtained for the studied years and stations in the Sado Estuary. The highest average diversity and evenness were obtained in 2019 at EM (2.11 and 0.55, respectively). However, the maximum diversity and evenness were recorded at MR during April 2018 (Shannon–Wiener diversity of 3.23 and Evenness of 0.83), when 49 taxonomic entities were identified, all present in low abundances (Table 2).

**Table 2:** Interannual variation of phytoplankton diversity in the different regions of Sado Estuary.

Station	Year	Nº of taxonomic entities		Abundance (10^3^cell L^−1^)		Diversity index (*H*′)		Evenness (*J*)	
		Total	Min–Max	Avg	Min–Max	Avg	Min–Max	Avg	Min–Max
AC	2018	66	16–32	**115**	10–**424**	1.31	0.30–2.12	0.42	0.09–0.64
	2019	65	17–35	46	11–261	1.98	0.86–2.64	0.61	0.24–0.79
MC	2018	84	17–39	74	12–394	1.25	0.77–1.72	0.38	0.23–0.55
	2019	79	20–46	40	12–101	1.74	0.79–2.42	0.51	0.24–0.70
MR	2018	110	24–49	37	96–80	1.84	0.60–**3.23**	0.50	0.19–**0.83**
	2019	87	27–46	54	12–154	1.71	1.13–2.28	0.48	0.32–0.66
EM	2018	**144**	34–**67**	86	14–259	2.02	0.22–2.89	0.52	0.06–0.70
	2019	134	45–53	39	14–91	**2.11**	1.00–2.70	**0.55**	0.26–0.70

Number of taxonomic entities: total of each year and minimum (Min) and maximum (Max) identified in one sample. Abundance (10^3^cell L^−1^), Shannon–Wiener diversity index (H’), and Evenness index (J): annual average (Avg) and minimum (Min) and maximum (Max) identified in one sample. Maxima values are shown in bold.

The PERMANOVA analysis of the phytoplankton assemblages indicated significant differences between years (p = 0.004, Table 3), sampling sites (p = 0.001, Table 3) and seasons (p = 0.001, Table 3). Since the results also indicated significant interactions between factors “Years $\times$ Season” (p = 0.023, Table 3) and “Site $\times$ Season” (p = 0.001, Table 3), pairwise comparisons are shown in Table 4. The “Years $\times$ Season” comparison only indicated differences between the years during summer (p = 0.003, Table 4). The “Site $\times$ Season” comparison did not indicated differences between AC/MC and MC/MR during spring, as well as for all the study site comparisons during winter (Table 4).

**Table 3:** Summary of results of permutational univariate analysis of variance (PERMANOVA) of the phytoplankton assemblages in the Sado Estuary for factors year, study site and season.

Source	df	SS	MS	Pseudo-F	P(perm)	perms
Year	1	2361	2361	3.03	**0.004**	996
Site	3	23,919	7973.1	10.232	**0.001**	999
Season	3	9566.8	3188.9	40.925	**0.001**	997
YearxSite	3	2901.2	967.08	12.411	0.179	997
YearxSeason	3	3987	1329	17.056	**0.023**	999
SitexSeason	9	11,625	1291.6	16.576	**0.001**	998
YearxSitexSeason	9	8368	929.78	11.932	0.116	996

“df”: degrees of freedom; “SS”: sum of squares; “MS”: mean squares; “Pseudo-F”: pseudo-F ratio; “P(perm)”: permutation P-value and “perms”: number of permutations. *p*-values smaller than 0.05 are presented in bold.

**Table 4:** Pairwise tests within the factors “Years $\times$ Season” (p = 0.023, Table 1) and “Site $\times$ Season”.

Groups	Spring		Summer		Autumn		Winter		
	P(perm)	perms	P(perm)	perms	P(perm)	perms	P(perm)	perms	P(MC)
2018, 2019	0.078	998	**0.003**	998	0.119	999	0.146	991	–
AC, MC	0.061	941	**0.001**	988	**0.004**	984	0.608	90	0.483
AC, MR	**0.004**	939	**0.001**	978	**0.004**	985	**0.033**	90	0.253
AC, EM	**0.001**	935	**0.001**	988	**0.001**	986	**0.019**	90	0.046
MC, MR	0.104	987	**0.002**	987	**0.006**	988	**0.045**	90	0.223
MC, EM	**0.003**	986	**0.001**	986	**0.001**	988	**0.035**	90	0.049
MR, EM	**0.022**	987	**0.003**	944	**0.001**	988	**0.009**	90	0.071

“P(perm)”: permutation P-value, “perms”: number of permutations and “P(MC)”: Monte-Carlo *p* value. *p*-values smaller than 0.05 are presented in bold.

**Figure 7:**
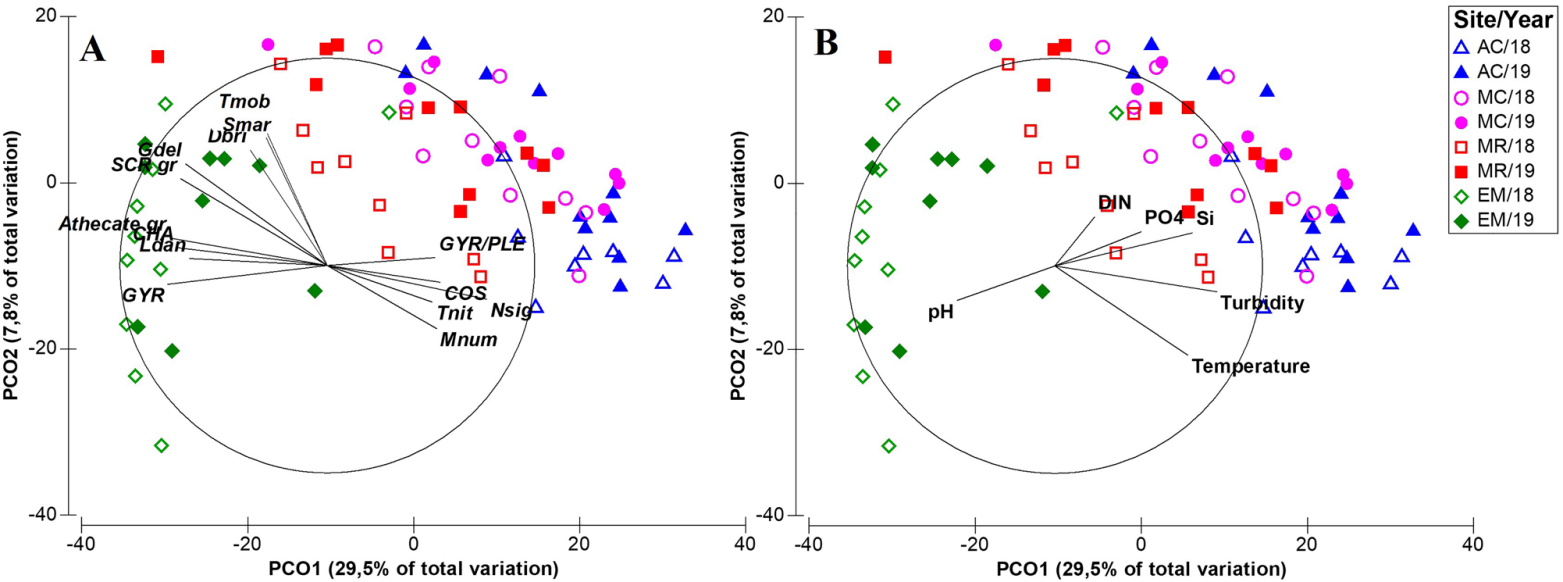
Principal coordinates analysis (PCO) plots of the abundance and composition of the phytoplankton community between each study site, based on a Bray–Curtis resemblance matrix. For each site, the symbols correspond to different sampling dates. (**A**)—some of the taxonomic entities (abbreviated according to Table S2) with correlations above 0.5 are represented as vectors overlapping the ordination, and (**B**)—physico-chemical variables with correlations above 0.2 with the PCO axes are represented as overlapping vectors.

**Figure 8:**
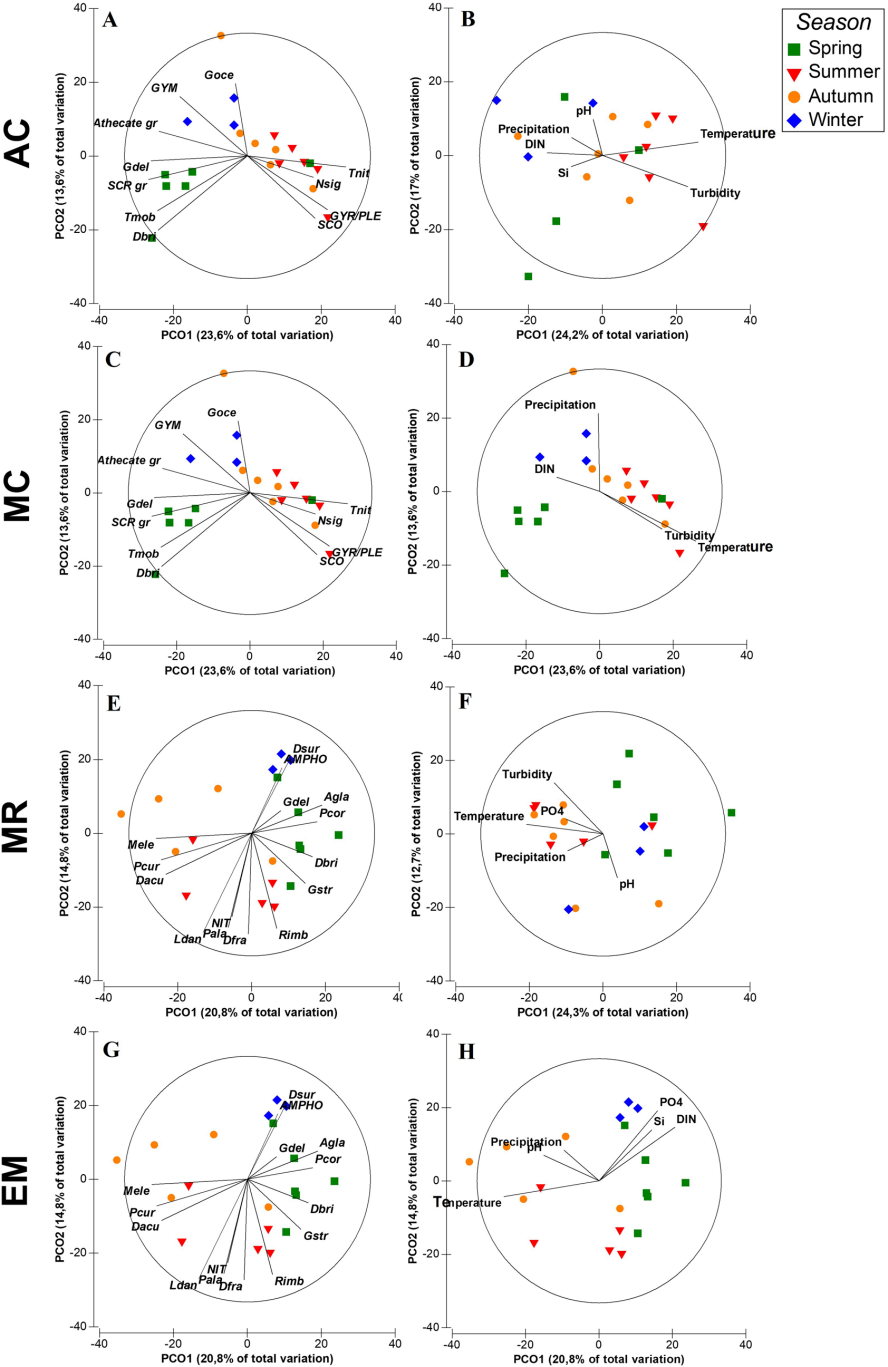
Principal coordinates analysis (PCO) plots of the abundance and composition of the phytoplankton community for each study site (**A**/**B**—AC,** C**/**D**—MC,** E**/**F**—MR and** G**/**H**—EM). Represented as vectors overlapping the ordination are some of the taxonomic entities (abbreviated according to Table S2) with correlations above 0.5 (left panel), and the physico-chemical variables with correlations above 0.2 (right panel).

PCO ordinations were used to analyze the spatial and temporal patterns of the phytoplankton assemblages (Figs. 7,8). Spatial patterns in the phytoplankton community composition were better observed when considering the axes 1 and 2 of the PCO analysis, with both axes explaining 37.3% of total phytoplankton variance (Fig. 7). The separation between the stations EM, MR and MC/AC can be observed along axis 1, while the separation between MC and AC occurred along axis 2. The results showed that a higher contribution of several chain-forming diatoms and dinoflagellates species were more related with the downstream station (EM) (e.g., the diatoms *Chaetoceros* spp., *Guinardia delicatula* and *Leptocylindrus* cf. *danicus*, and the dinoflagellates *Gyrodinium* spp., *Scrippsiella* group and athecate unidentified species) (Fig. 7A). These assemblages appeared to be more correlated with higher pH levels (Fig. 7B). In upstream stations (AC and MC), phytoplankton assemblages were dominated by several pennate diatom species (e.g., *Nitzschia* cf. *sigma*, *Gyrosigma* spp.*/Pleurosigma* spp.) and by some centric diatoms (e.g., *Coscinodiscus* spp. and *Melosira nummuloides*) (Fig. 7A). This phytoplankton assemblage was correlated with warmer waters, richer in nutrients and with higher turbidity levels (Fig. 7B). The station located in the middle of the estuary (MR) showed higher similarity with phytoplankton assemblages recorded upstream (Fig. 7).

This PCO also showed that the phytoplankton community composition patterns observed between the years (2018 and 2019) were less evident than the spatial patterns (Fig. 7). To better identify the taxonomic entities responsible for the phytoplankton community structure between the factor Year, the SIMPER analysis was performed (Table S3). The average dissimilarity observed on phytoplankton community between 2018 and 2019 (51.7%) resulted from the overall assemblage, with each species contributing for a small percentage of the dissimilarity between both years. Some of the taxonomic entities that contributed most for the dissimilarity between the years were small unidentified flagellates (< 15 µm), *G. delicatula*, *Thalassiosira* spp. (< 10 µm), *Skeletonema marinoi*, *Thalassionema nitzschioides*, *Chaetoceros* spp. and *L.* cf. *danicus* (Table S3).

PCO analyses conducted for each sampling site showed the seasonal patterns in the phytoplankton communities at each location (Fig. 8), with the first two axes explaining 41.2%, 37.2%, 37% and 35.6% of total phytoplankton variance, for stations AC, MC, MR and EM, respectively. At AC there was a higher correlation of winter/spring phytoplankton assemblages with drivers such as nutrient concentrations (DIN and Si) and precipitation (Fig. 8A). During winter and spring, phytoplankton assemblages had a high contribution of athecate unidentified dinoflagellates and the diatom *S. marinoi*. During winter, cryptophytes and dinoflagellates belonging to the group *Heterocapsa* spp.*/Azadinium* spp. were also important, while in spring there was a higher correlation with the dinoflagellates of *Scrippsiella* group and with several chain-forming diatoms species (e.g., *Thalassiosira* spp. (< 10 µm)—Fig. 8A, G.*. delicatula* and *Rhizosolenia* cf. *imbricate*—not shown). Summer/autumn phytoplankton community composition had a higher correlation with higher temperature and turbidity levels (Fig. 8A). During summer, there was a higher correlation with several pennate diatoms (e.g., *T. nitzschioides, Diploneis* cf. *bombus,* cf. *Scoliotropis* spp. and *Cylindrotheca closterium*). In autumn dominated a mixed community of pennate diatoms (e.g., *C. closterium* and unidentified species > 80 µm) and small dinoflagellates (e.g., *Heterocapsa* spp.*/Azadinium* spp. and unidentified species < 15 µm) (Fig. 8A).

At MC the explanatory physico-chemical variables were almost the same that were found in AC, although with precipitation having a higher correlation with winter samples (Fig. 8B). The winter phytoplankton community was better correlated with *Gymnodinium* spp. and other athecate unidentified dinoflagellates, as well as with the diatom *Grammatophora oceanica* (Fig. 8B). Spring had a higher contribution of the dinoflagellates belonging to the *Scrippsiella* group and several diatoms, such as *Trieres mobiliensis, Ditylum brightwellii* and the chain-forming *G. delicatula* (Fig. 8B). The summer/autumn phytoplankton assemblage had a higher contribution of several pennate diatoms, most of them benthic species (e.g., *Gyrosigma* spp.*/Pleurosigma* spp., cf. *Scoliotropis* spp. and *N.* cf. *sigma*) (Fig. 8B).

At MR, turbidity and temperature were again highly correlated with summer/autumn phytoplankton assemblages, but in this location there was also a correlation of 0.30 with precipitation (especially recorded during autumn, cf. Figure 3) and with phosphate concentration (Fig. 8C). In terms of phytoplankton assemblages, it was possible to distinguish two different groups, the winter/spring and the summer/autumn communities. The dinoflagellate *Prorocentrum cordatum* and several diatom species (e.g., *D. brightwellii, G. delicatula, G. striata* and *Asterionellopsis glacialis*) had higher correlation with spring communities, and the diatoms of the centric group (20–40 µm) and *S. marinoi* with the winter assemblage (Fig. 8C). The summer and autumn phytoplankton assemblages had a higher contribution of pennate diatoms (e.g., *N.* cf. *sigma, Bacillaria paxillifera*), dinoflagellates (e.g., *Karenia* spp. (< 20 µm), *Dinophysis caudata* and *Prorocentrum micans*) and the chlorophyte *Monoraphidium* cf. *griffithii*.

At EM, higher nutrient concentrations were most correlated with winter and some spring samples, temperature with summer and autumn samples, and precipitation and pH mainly with autumn samples (Fig. 8D). At this station, winter phytoplankton community composition had a high correlation with pennate diatoms, such as *Amphora* spp. and cf. *Delphineis surirella*. In spring, a community similar to that recorded in MR was found (dinoflagellate *P. cordatum* and several chain-forming diatoms species (e.g., *D. brightwellii, G. delicatula, G. striata* and *A. glacialis*). Summer and autumn were mainly correlated with high abundance of several dinoflagellate species (e.g., *Dinophysis acuta, Protoperidinium* cf. *curtipes*—Fig. 8D, *Torodinium robustum, D. caudata, P. micans*—not shown). During summer, a mixed community of dinoflagellates was found together with diatoms (e.g., *R.* cf. *imbricata, Proboscia alata, Nitzschia* spp., *L.* cf. *danicus*), and during autumn there was also a high correlation with the cyanobacteria *Merismopedia* cf. *elegans* (Fig. 8D).

## Discussion

The present study provides insights to the understanding of phytoplankton assemblage structure, variability, and dynamics in well-mixed estuaries. The Sado Estuary revealed significant differences in the phytoplankton communities at different estuarine regions and seasons, with different drivers shaping the assemblages. These differences will be discussed in the following sections.

### Phytoplankton spatial variability and main physico-chemical drivers

The phytoplankton dynamics in the Sado Estuary were characterized by several spatial differences. Chlorophyll *a* concentration was always higher upstream (maximum of 12 mg m^−3^) than in the middle and outer regions, a pattern that is in accordance with most of the studies conducted in the Sado Estuary^9,17,27,42^, as well as in other temperate estuarine systems^23,25,43,44^. However, higher Chl *a* concentration can occur at the Alcácer channel, as observed in a previous study (25 mg m^−3^ average in the 1990’s^22^). Still, the present results agree with the current classification of Sado Estuary of High ecological condition based on the phytoplankton biomass indicator, as previously reported^9,10^ This classification is based on the national methodology for the assessment of ecological quality, as described by Brito et al. ^9,10^.

In this study, the phytoplankton cell abundances were always below 500 × 10^3^ cell L^−1^, which is much lower than the maximum measured in 1992 for the same region, Alcácer channel (5300 × 10^3^ cell L^−1^)^22^. The phytoplankton abundances obtained in this study were also much lower than the thresholds established within the framework of the WFD for the occurrence of a phytoplankton ‘bloom’^9,10^. This result suggests that no significant phytoplankton blooms occurred in the Sado Estuary, during the studied period. Further research is still needed to fully understand the cause of this observed change. Nevertheless, it could be hypothesized that the decrease in phytoplankton biomass and abundances in the last two decades could be related to the recovery of the shellfish biomass in the Sado Estuary, particularly the oyster banks. Those banks, which occupied thousands of hectares before 1970’s, were confined to few examples in the Alcácer channel area during the 1990’s period^45^. From the 2000s onwards the recovery of those banks has been reported^46,47^. The expansion of the Japanese clam (*Ruditapes philippinarum*) in the estuary was also observed in recent years, and this increasing trend is expected to continue in the future, as observed in the Tagus Estuary ^48,49^, one of the largest estuaries on the west coast of Europe, located around 40 km northwest of the Sado Estuary. In fact, a previous study conducted in the Tagus Estuary also reported lower chlorophyll *a* concentrations after 2004 compared to the ones obtained in the 1980s, and discussed the increase in the invasive Japanese clams as a possible cause^43,50^.

**Figure 9:**
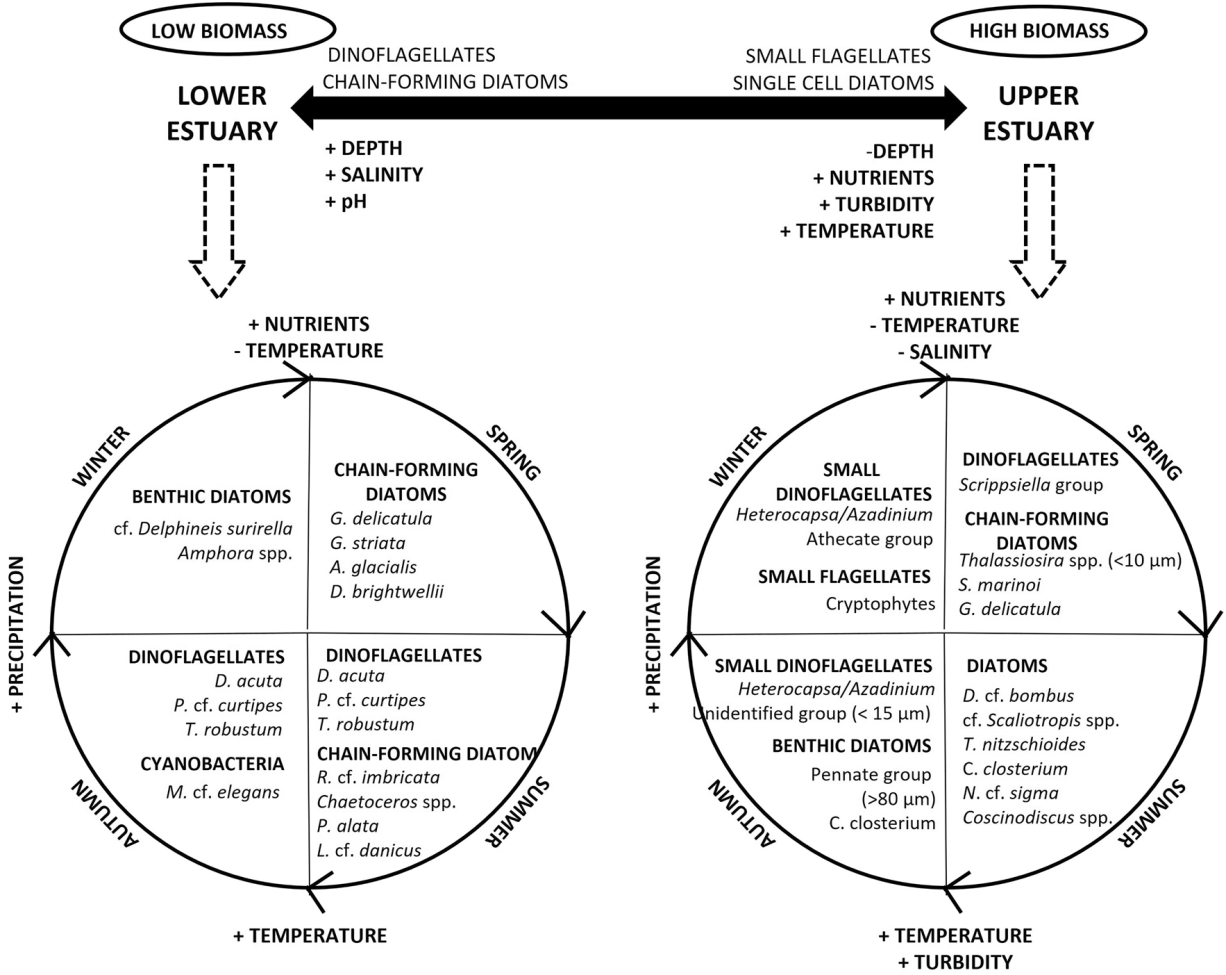
Summary diagram of the dynamics of the phytoplankton community in the Sado Estuary. The main spatial and temporal differences are shown.

Three phytoplankton taxonomic groups were highlighted as being of high importance in the composition of the phytoplankton assemblages in the different regions of the Sado Estuary: diatoms, cryptophytes, and dinoflagellates. Diatoms were the dominant group, favored by the well-mixed nature of the estuary^15^. Diatoms dominance is a common pattern in this estuary and in other estuarine systems^18,22,44,51,52^. This study showed that several diatom species dominated the stations located in the inner region, with a high contribution of benthic/epibenthic species (e.g., *Gyrosigma/Pleurosigma* group, *Nitzschia sigma* and *Melosira nummuloides*), while several chain-forming diatom species dominated near the estuary mouth (e.g., *Chaetoceros* spp., *Leptocylindrus danicus* and *Guinardia delicatula*). The upper estuary has shallower waters and high turbidity due to the high tidal mixing^27^, conditions that favor the development and resuspension of benthic diatoms. On the other hand, deeper, clearer, and saltier waters characterized the estuary mouth. These conditions, together with the entry of coastal phytoplankton communities during tidal intrusion, favored the dominance of chain-forming diatoms in the outermost estuarine region (cf. Fig. 9).

Cryptophytes were the second most prevalent group observed in the entire estuary, reaching higher abundances in the inner regions, suggesting the presence of a local fast-growing cryptophyte community all year round in this estuary. Within the estuary, cryptophytes were recorded in abundances two to three times higher than diatoms. A previous study which occurred in this estuary in 1992–1993^22^, reported higher abundances of cryptophytes in the inner regions, but not as prevalent as in 2018–2019. In fact, Coutinho^22^ reported cryptophytes abundances that were four to six times lower than diatoms. The increase of cryptophytes in the Sado Estuary, associated with a decrease in diatoms is aligned with previous studies conducted in the Tagus Estuary^43^. The change of a diatom-dominant community to small flagellates’ dominance has also been suggested in other estuarine systems^43,50,53^. The main reason for the increase of cryptophyte dominance in estuaries is not fully understood but shellfish grazing and the increase in nutrients have been proposed as potential responsible factors^43,50^. Several factors have been pointed to be responsible for changes in phytoplankton composition in the aquatic ecosystems, namely: (i) alterations in nutrient ratios^54^; (ii) seasonality of the environmental variables^55^; (iii) introduction of non-indigenous herbivores^56^; and (iv) climate change effects^57^. Nutrient patterns in the Sado Estuary and their potential relationship with cryptophytes increase will be discussed in the following paragraphs.

Higher DIN, phosphate and silicate concentrations have been reported in the inner regions of the Sado Estuary, than in the lower estuary^21,22,27^. This pattern was also observed in the present work. In addition, near the estuary mouth, silicate was frequently observed to be below the accepted standard molar ratios that favored phytoplankton growth (Si:N:P = 16:16:1). It was also mainly near the estuary mouth that the nutrient concentrations were recorded several times below the half-saturation constants reported for phytoplankton growth in estuarine and coastal systems (*Km* = 1 µmol/L for N uptake, *Km* = 0.2 µmol/L for P uptake and *Km* = 2 µmol/L for Si uptake)^58^. The half-saturation constant represents the concentration at which nutrient uptake is half of its maximum value. Concentrations below these values are associated with greatly reduced uptake rates, which could be enough to limit phytoplankton growth rates^59^. Nutrient availability in the Sado Estuary during the present work suggests that nutrients were not a key limiting factor for phytoplankton growth in the inner stations but may have affected phytoplankton growth in the outer station. Nevertheless, it is important to highlight that there is a great inter-annual variability in nutrient concentrations in the Sado Estuary, as we can see in the present work and in the study performed 25 years ago^22^. Nevertheless, additional data with higher temporal resolution are needed to discuss potential changes in nutrient concentrations and nutrient ratios.

It is known that cryptophytes are most competitive under nonlimiting nutrient conditions^60^, taking advantage of waters enriched in nitrates, ammonia, and phosphate with both natural and anthropogenic origins^61^. Different preferences or uptake rates of nitrogen sources have also an important role in the phytoplankton dominant groups. While diatoms, along with the silicate, preferably make use of nitrate, cryptophytes have a higher advantage in ammonium enriched waters^62^. Actually, a previous study conducted in this estuary during the same period of investigation reported higher ammonium concentrations in the inner region of the estuary than in the outer region^27^, which may have also favored the high cryptophytes abundance observed here. In addition, since cryptophytes are small-sized and motile cells, this may also give an advantage in high turbidity regions (light-limited)^52^ and a more efficient nutrient uptake when compared to large non-motile cells such as diatoms^63^.

Lastly, dinoflagellates were the third group that stood out in the present work (e.g., *Scrippsiella* and athecate groups), with sporadic dominance and with a preference for occurrence downstream. An increase in dinoflagellate abundances and in species number, especially near the estuary mouth, was observed here when compared with the 1990s observations^22^. Dinoflagellates are usually highly diverse and abundant in the coastal region^55,64^. Since the present study sampling was conducted during high tide, transport of this group is likely to occur from the nearby coastal region to the estuary, losing intensity from downstream to upstream regions. On the other hand, the study conducted in the 1990s covered low tide conditions, what could partially explain the lower abundance and diversity of dinoflagellates in the Sado Estuary, particularly near the estuary mouth. The differences observed between these studies highlight the importance of investigating the variability of phytoplankton communities under different tide regimes.

### Phytoplankton temporal variability and main physico-chemical drivers

Inter-annual differences in phytoplankton were observed in this study, namely higher phytoplankton abundance and biomass during 2018. The year 2018 was characterized by having higher nutrient concentrations than 2019, especially during spring, when favored phytoplankton growth in that year. Lower salinity and higher CDOM also characterized the spring of 2018, especially in the inner regions of the estuary. In fact, the sampling performed in May was conducted under heavy rain. Salinity and CDOM obtained during the winter/spring of 2018 reflected the increased rainfall, reinforcing the link between rainfall and terrestrial inputs. A previous study which occurred during the same period reported that higher wind intensities in the spring/summer of 2018 together with tides and currents may have also caused bottom resuspension, particularly in the inner region characterized by low water depth^26^. These hydrodynamic conditions contributed to higher turbidity and a substantial increase in CDOM^26^. All these factors may have favored the higher abundance and biomass of phytoplankton in spring and in summer, especially during 2018. This pattern of maxima phytoplankton abundance and biomass between spring and summer is in accordance with the seasonal phytoplankton pattern previously observed in this estuary^22,26^ and in other coastal and estuarine systems^24,43,65^.

It is known that phytoplankton blooms can be classified as recurrent seasonal events that may persist for weeks’^66^, or as shorter episodic events with some algal blooms reaching blooming conditions and starting to disappear within one week^67^. Since the scope of this study was to evaluate the seasonal phytoplankton variability and dynamics in the Sado Estuary, monthly sampling used here seems to be suitable. Monthly periodicity sampling was considered as high frequency sampling in previous phytoplankton studies^53^.

This study highlights several seasonal significant different patterns in phytoplankton assemblages that characterize the upper and the lower estuarine regions (cf. Fig. 9). The upper estuary was characterized by the presence of three different diatoms assemblages: (i) chain-forming diatom species, most of them of small size (e.g., the centric diatoms *Thalassiosira* spp. < 10 µm and *Skeletonema marinoi*), dominated in the nutrient richer spring waters; (ii) several single-cell species (e.g., *Diploneis* cf. *bombus, Cylindrotheca closterium, Nitzschia* cf. *sigma* and *Coscinodiscus* spp.) dominated in the turbid and warmer summer waters; and (iii) other diatoms, mostly of the benthic group, dominated in autumn (e.g., pennate group > 80 µm and *Cylindrotheca closterium*). In the higher rainfall seasons (autumn and winter), dinoflagellates of small size (< 15 µm) and other small flagellates (e.g., cryptophytes) were relevant to the phytoplankton assemblage, the latter with a higher contribution in winter. Overall, in the upper estuary, the phytoplankton community composition was dominated by cryptophytes, by small centric diatoms and by benthic diatom species. These are all groups with high photosynthetic efficiency in low light/high turbidity conditions, as previously reported in other estuarine systems^52^.

In the lower estuary, three diatoms assemblages were also highlighted, although different from the upper estuary (cf. Fig. 9): (i) benthic diatoms were essentially found during winter (e.g., cf. *Delphineis surirella* and *Amphora* spp.); (ii) chain-forming diatoms dominated in nutrient-rich spring waters (e.g., *Guinardia* spp., *Asterionellopsis glacialis* and *Ditylum brightwellii*); and iii) other chain-forming diatoms species (e.g., *Rhizosolenia* cf. *imbricata* and *Proboscia alata*), typically associated with summer stratification^55,64^, characterized the phytoplankton community in summer. In addition to diatoms, the other phytoplankton group that contributed the most for the phytoplankton community abundance in this region was the dinoflagellates (e.g., *Dinophysis* spp., *Protoperidinium* spp. and *Torodinium robustum*). This group, although reaching sporadically high concentrations during spring (April 2018), was dominant in the warmer seasons. In estuaries, dinoflagellates occur preferably in periods characterized by high residence time, like summer and autumn^24,68^. In the outer estuarine region, the influence of the phytoplankton developed in the nearby coastal region is also expected, namely dinoflagellates and diatoms. These phytoplankton groups are favored, respectively, by coastal thermal stratification and upwelling conditions^55^.

During the present study, two dinoflagellate blooms occurred near the estuary mouth in April and October 2018, with cell abundances accounting for more than 90% of the total phytoplankton. These two blooms were associated with the presence of harmful algal bloom species (HABs), respectively, *Prorocentrum cordatum* and *Gymnodinium catenatum* (together with *Gymnodinium* spp.). Throughout the study, these blooms were the only ones that raised concern for the occurrence of harmful algal species in the Sado Estuary, even if restricted to the lower region. This result agrees with previous studies conducted in this estuary that reported the occurrence of harmful dinoflagellates as uncommon and always present in small concentrations, except for *P. cordatum,* known as reaching potentially high abundances^22^. The present work reported, for the first time, a bloom of *G. catenatum* inside the Sado Estuary, reaching abundances above the interdiction reference levels (> 1500 cell L^−1^)^69^. This bloom coincided with the proliferation of *G. catenatum* that occurred in the west Iberian coast, between Peniche and Sagres, which contaminated bivalves above the regulatory levels and caused severe neurological symptoms in two consumers^70^.

## Final considerations

Understanding the relationship between phytoplankton assemblages and underlying environmental drivers is essential for the management of estuarine systems. In the Sado Estuary, seasonal phytoplankton blooms occurred in the entire system with biomass concentrations that agree with the ‘High’ ecological status classification. This estuary was characterized by significant differences on phytoplankton assemblages in different regions and seasons. Local physico-chemical processes, such as water temperature, turbidity, nutrient concentrations, and salinity explained most of the spatial and temporal phytoplankton variability (Fig. 9).

Small phytoplankton cells, belonging to different taxonomic groups, dominated in the shallow and turbid estuarine regions, showing high photosynthetic efficiency. Several chain-forming diatom species and dinoflagellates dominated in the deeper and saltier estuary mouth, revealing a high influence of coastal phytoplankton communities and dynamics in the lower estuary during high tide period. The occurrence of HABs in the Sado Estuary is sporadic and apparently restricted to the estuary mouth. This suggests that the inner regions of the estuary are suitable for aquaculture production. Nevertheless, information on HABs with high temporal resolution can help producers to better manage the growth and harvesting of bivalves. It can also be key for an effective selection of the production sites.

This study highlights the apparent changes in the dominant phytoplankton groups in the last two decades, such as an increase in cryptophytes over diatoms in the inner estuarine regions, and an increase in dinoflagellates near the estuary mouth. In the future, it is important to assess the variability of phytoplankton communities under different tide regimes (high vs low tide), as well as during fortnight tidal cycle. This study can be also an important contribution to the development of novel classification methods (under the scope of the WFD) based on the phytoplankton community structure. The development of this method will allow not only to further evaluate ecosystem health and its functioning, as well as to contribute to the protection of natural capital and ecosystem services provided by the estuary.

## Supplementary Information

Supplementary Information.

## Data Availability

The datasets used and/or analyzed during the current study available from the corresponding author on reasonable request.

## References

[1] Barbier EB (2011). The value of estuarine and coastal ecosystem services. Ecol. Monogr..

[2] Kennish MJ (2002). Environmental threats and environmental future of estuaries. Environ. Conserv..

[3] Brettum, P. & Andersen, T. *The Use of Phytoplankton as Indicators of Water Quality.* Norwegian Institute for Water Research SNO Report 4818 (2005).

[4] Anderson DM, Glibert PM, Burkholder JM (2002). Harmful algal blooms and eutrophication nutrient sources, composition, and consequences. Estuaries.

[5] Glibert PM (2010). Modeling of HABs and eutrophication: Status, advances, challenges. J. Mar. Syst..

[6] Reynolds CS (2006). The Ecology of Phytoplankton.

[7] European Commission. *Directive 2000/60/EC of the European Parliament and of the Council of 23 October 2000 on establishing a framework for community action in the field of water policy*. (2000).

[8] Carletti, A., & Heiskanen, A.-S. *Water Framework Directive intercalibration technical report-Part 3: Coastal and Transitional Waters*. Office for Official Publications of the European Community (2009).

[9] Brito AC (2012). Defining phytoplankton class boundaries in Portuguese transitional waters: An evaluation of the ecological quality status according to the Water Framework Directive. Ecol. Indic..

[10] Brito AC (2020). Integrating in situ and ocean color data to evaluate ecological quality under the water framework directive. Water (Basel).

[11] Rocha CP, Cabral HN, Marques JC, Gonçalves AMM (2022). A global overview of aquaculture food production with a focus on the activity’s development in transitional systems—The case study of a South European Country (Portugal). J. Mar. Sci. Eng..

[12] Freitas MC (2008). Anthropogenic influence in the Sado estuary (Portugal): a geochemical approach Influencia antrópica en el estuario de Sado (Portugal): Una aproximación geoquímica. J. Iber. Geol..

[13] Martins F, Leitão P, Silva A, Neves R (2001). 3D modelling in the Sado estuary using a new generic vertical discretization approach. Oceanol. Acta.

[14] Ferreira, J. G. *et al. Identification of sensitive areas and vulnerable zones in transitional and coastal portuguese systems: application of the United States National Estuarine Eutrophication Assessment to the Minho, Lima, Douro, Ria de Aveiro, Mondego, Tagus, Sado, Mira, Ria Formosa and Guadiana systems.* (2003).

[15] Biguino, B., Sousa, F. & Brito, A. C. Variability of currents and water column structure in a temperate estuarine system (Sado estuary, Portugal). *Water (Basel)* **13**, 187 (2021).

[16] Largier, J. Low-Inflow estuaries: Hypersaline, inverse, and thermal scenarios. in *Contemporary Issues in Estuarine Physics* 247–272 (2010).

[17] Silva, E. S., Assis, M. E. & Sampayo, M. A. M. *Primary Productivity in the Tagus and Sado Estuaries from May 1967 to May 1968*. Notas e Estudos do Instituto de Biologia Marítima., Nº 37 (1969). (*in Portuguese*)

[18] Sampayo, M. A. M. *Bacillariophyceae do Estuário do Sado. Estudo Qualitativo e Quantitativo; Variações sazonais*. Notas e Estudos do Instituto de Biologia Marítima, nº 37 (1970). (*in Portuguese*)

[19] Oliveira, M. R. & Coutinho, M. T. P. *Estado trófico e dinâmica do fitoplâncton das zonas superior, média e inferior do Estuário do Sado*. Relatórios Técnico Científicos INIP 59 (1992). (*in Portuguese*)

[20] Coutinho, M. T. P. *Variação Espacio-Temporal do Fitoplâncton no Estuário do Sado*. Seminário sobre Recursos Haliêuticos, Ambiente, Aquacultura e Qualidade do Pescado da Península de Setúbal. Publicações avulsas do IPIMAR, Nº 1 (1994). (*in Portuguese*)

[21] Cabeçadas G, Nogueira M, Brogueira MJ (1999). Nutrient dynamics and productivity in three European Estuaries. Mar. Pollut. Bull..

[22] Coutinho, M. T. P. Comunidade fitoplantónica do Estuário do Sado. Estrutura, Dinâmica e Aspectos Ecológicos. Dissertação para Investigador Auxiliar. INIAP/IPIMAR (2003). (*in Portuguese*)

[23] Seoane S, Laza A, Urrutxurtu I, Orive E (2005). Phytoplankton assemblages and their dominant pigments in the Nervion River estuary. Hydrobiologia.

[24] Gameiro C, Cartaxana P, Brotas V (2007). Environmental drivers of phytoplankton distribution and composition in Tagus Estuary, Portugal. Estuar Coast Shelf. Sci..

[25] Popovich CA, Marcovecchio JE (2008). Spatial and temporal variability of phytoplankton and environmental factors in a temperate estuary of South America (Atlantic coast, Argentina). Cont Shelf Res..

[26] Sent G (2021). Deriving water quality parameters using sentinel-2 imagery: A case study in the Sado Estuary, Portugal. Remote Sens. (Basel).

[27] Nascimento Â (2021). Tidal variability of water quality parameters in a mesotidal estuary (Sado Estuary, Portugal). Sci. Rep..

[28] Gonçalves C, Brogueira MJ, Nogueira M (2015). Tidal and spatial variability of nitrous oxide (N2O) in Sado estuary (Portugal). Estuar Coast Shelf Sci..

[29] Jeffrey SW, Humphrey GF (1975). New spectrophotometric equations for determining chlorophylls a, b, c1 and c2 in higher plants, algae and natural phytoplankton. Biochem. Physiol. Pflanz..

[30] Throndsen, J. Preservation and storage. in *Phytoplankton Manual: Monographs on Oceanographic Methodology 6* 69–74 (UNESCO, 1978).

[31] Utermöhl H (1958). Zur Ver vollkommung der quantitativen phytoplankton-methodik. Mitteilungen der internationale Vereinigung für theoretische und angewandte Limnologie.

[32] Dodge, J. D. *Marine Dinoflagellates of the British Isles.* H.M. Stationery Office (1982).

[33] Hoppenrath, M., Elbrächter, M. & Drebes, G. *Marine phytoplankton. Selected microphytoplankton species from the North Sea around Helgoland and Sylt.* E. Schweizerbart’sche Verlagsbuchhandlung, (2009).

[34] Peragallo, H. & Peragallo, M. *Diatomées marines de France.* Grez-surLoing (1908).

[35] Schiller, J. *Dinoflagellatae*. vols I and II. Verlagsgeselleschaft M.B.H. (1937).

[36] Thomas, C. R. *Identifying Marine Phytoplankton*. Academic Press (1997).

[37] Guiry, M. D. & Guiry, G. M. AlgaeBase. *World-wide electronic publication, National University of Ireland, Galway.* (2020). https://www.algaebase.org (searched on 28 January 2020).

[38] Clarke, K. R. & Gorley, R. N. PRIMER v6: User Manual/Tutorial (Plymouth Routines in Multivariate Ecological Research). *PRIMER-E, Plymouth UK* 192 (2006). 10.1111/j.1442-9993.1993.tb00438.x.

[39] Anderson, M. J., Gorley, R. N. & Clarke, K. R. PERMANOVA + for PRIMER: Guide to Software and Statistical Methods. *PRIMER-E, Plymouth* 214 (2008).

[40] Redfield A. C., Ketchum B. H. & Richards, F. A. The influence of organisms in the composition of seawater. in *The Sea* (ed. Hill, M. N.) 26–77. Wiley (1963).

[41] Brzezinski MA (1985). The Si: C: N ratio of marine diatoms: interspecific variability and the effect of some environmental variables 1. J. Phycol..

[42] Cabeçadas, L. *Contribuição para o estudo do ciclo anual do fitoplâncton do estuário do Sado.* Relatório interno, Secretaria de Estado do Ambiente (1980). (*in Portuguese*)

[43] Brito AC (2015). Changes in the Phytoplankton Composition in a Temperate Estuarine System (1960 to 2010). Estuaries Coasts.

[44] Cereja R, Brotas V, Cruz JPC, Rodrigues M, Brito AC (2021). Tidal and physicochemical effects on phytoplankton community variability at Tagus Estuary (Portugal). Front. Mar. Sci..

[45] Ruano F (1994). Contribuição para o apoio à moluscicultura no Estuário do Sado e Lagoa de Albufeira - Seminário sobre Recursos Haliêuticos, Ambiente, Aquacultura e Qualidade do Pescado da península de Setúbal. Publicações avulsas do IPIMAR.

[46] Cabral, H. *et al. Estado atual da ostra-portuguesa (Crassostrea angulata) no estuário do Sado, ameaças e oportunidades para a sua exploração como recurso - CRASSOSADO - Relatório Final*. (2016). (*in Portuguese*)

[47] Brito, A. C. *et al. Promover a aquacultura sustentável no Estuário do Sado - AQUASADO – Relatório Final*. (2022). (*in Portuguese*)

[48] Coelho P, Carvalho F, Goulding T, Chainho P, Guerreiro J (2021). Management Models of the Manila Clam (Ruditapes philippinarum) Fisheries in Invaded European Coastal Systems. Front. Mar. Sci..

[49] Cabral, S. *et al.* Non-indigenous species in soft-sediments: Are some estuaries more invaded than others? *Ecol Indic* **110** (2020).

[50] Cloern JE (2001). Our evolving conceptual model of the coastal eutrophication problem. Mar. Ecol. Prog. Ser..

[51] Rocha C, Galvão H, Barbosa A (2002). Role of transient silicon limitation in the development of cyanobacteria blooms in the Guadiana estuary, southwestern Iberia. Mar. Ecol. Prog. Ser..

[52] Gameiro C, Zwolinski J, Brotas V (2011). Light control on phytoplankton production in a shallow and turbid estuarine system. Hydrobiologia.

[53] Wilson JR, Wilkerson FP, Blaser SB, Nielsen KJ (2021). Phytoplankton community structure in a seasonal low-inflow estuary adjacent to coastal upwelling (Drakes Estero, CA, USA). Estuaries Coasts.

[54] Ferreira A (2020). Phytoplankton response to nutrient pulses in an upwelling system assessed through a microcosm experiment (Algarrobo Bay, Chile). Ocean Coast Manag..

[55] Santos M, Moita MT, Oliveira PB, Amorim A (2021). Phytoplankton communities in two wide-open bays in the Iberian upwelling system. J. Sea Res..

[56] Alpine, A. E. & Cloern, J. E. Trophic interactions and direct physical effects control phytoplankton biomass and production in an estuary. *Limnol. Oceanogr* **37** (1992).

[57] Cloern JE (2005). Climate anomalies generate an exceptional dinoflagellate bloom in San Francisco Bay. Geophys Res Lett.

[58] Dortch Q, Whitledget TE (1992). Does nitrogen or silicon limit phytoplankton production in the Mississippi River plume and nearby regions?. Cont Shelf Res..

[59] Fisher TR, Harding LW, Stanley DW, Ward LG (1988). Phytoplankton, Nutrients, and Turbidity in the Chesapeake, Delaware, and Hudson Estuaries. Estuar. Coast Shelf Sci..

[60] Klaveness D (1989). Biology and Ecology of the Cryptophyceae: Status and Challenges. Biol. Oceanogr..

[61] Seoane S (2011). Phytoplankton pigments and epifluorescence microscopy as tools for ecological status assessment in coastal and estuarine waters, within the Water Framework Directive. Mar Pollut Bull.

[62] Horner Rosser, S. M. J. & Thompson, P. A. Phytoplankton of the Swan - Canning Estuary: A comparison of nitrogen uptake by different bloom assemblages. *Hydrol Process* **15**, 2579–2594 (2001).

[63] Maranón E, Fernandez E (1995). Changes in phytoplankton ecophysiology across a coastal upwelling front. J Plankton Res.

[64] Moita, M. T. Estrutura , Variabilidade e Dinâmica do Fitoplâncton na Costa de Portugal Continental. Universidade de Lisboa (2001). (*in Portuguese*). https://www.ipma.pt/pt/publicacoes/pescas/index.jsp?page=teses.xml.

[65] Santos M (2020). Characterizing phytoplankton biomass seasonal cycles in two NE Atlantic coastal bays. Cont Shelf Res.

[66] Cloern JE (1996). Phytoplankton bloom dynamics in coastal ecosystems: A review with some general lessons from sustained investigation of San Francisco Bay, California. Rev. Geophys..

[67] Dubelaar GBJ, Geerders PJF, Jonker RR (2004). High frequency monitoring reveals phytoplankton dynamics. J. Environ. Monit..

[68] Brito AC (2022). Increased oyster aquaculture in the Sado Estuary (Portugal): How to ensure ecosystem sustainability?. Sci. Total Environ.

[69] IPMA, I. P. Instituto Português do Mar e de Atmosfera, I.P. (2021). http://www.ipma.pt/pt/bivalves (searched on 8 April 2022).

[70] Carvalho IL (2019). Paralytic shellfsh poisoning due to ingestion of contaminated mussels: a 2018 case report in Caparica (Portugal). Toxicon X.

[71] R Core Team. R: A language and environment for statistical computing. Preprint at (2022).

[72] Ferreira, J. G. *et al. Water Framework Directive-Transitional and Coastal Waters Proposal for the definition of water bodies*. (2005).

