# Supplementary material for: Spatio-temporal dynamics of phytoplankton community in a well-mixed temperate estuary (Sado Estuary, Portugal)
Source: Sci Rep. 2022 Sep 30;12:16423. doi: 10.1038/s41598-022-20792-6 (PMC9525256; doi:10.1038/s41598-022-20792-6)
Supplement: Supplementary file 1 — Supplementary Information. [file 41598_2022_20792_MOESM1_ESM.docx]

Table S1 **-** Geographical coordinates and maximum depth (m) of the sampling stations: AC – Alcácer channel, MC – Marateca channel, MR – Middle region, and EM – Estuary mouth.

| **Sampling Station** | **Latitude (ºN)** | **Longitude (ºW)** | **Maximum depth (m)** |
| --- | --- | --- | --- |
| AC | 38.415219 | 8.671550 | 6 |
| MC | 38.500000 | 8.748331 | 7 |
| MR | 38.470831 | 8.793889 | 20 |
| EM | 38.486669 | 8.930000 | 40 |

Table S2 **-** List of phytoplankton taxonomic entities identified in the Sado Estuary and maxima (Max) cell concentration in each study site (in cell L^-1^). Potentially harmful taxa are pointed with *. The corresponding abbreviation (Abbrev.) considered to the multivariate analyses is also shown.

| **TAXONOMIC ENTITIE** | **Abbrev.** | **Max (cell L^-1^)** | | | |
| --- | --- | --- | --- | --- | --- |
|  |  | **AC** | **MC** | **MR** | **EM** |
| **BACILLARIOPHYCEAE** |  |  |  |  |  |
| *Achnanthes longipes* |  | 120 | 0 | 0 | 0 |
| *Achnanthes* spp. |  | 0 | 600 | 20 | 0 |
| *Amphiprora* spp. |  | 0 | 40 | 0 | 0 |
| *Amphora* spp. | AMPHO | 22 | 40 | 20 | 40 |
| *Asterionellopsis glacialis* | Agla | 200 | 1040 | 1320 | 3540 |
| *Asteromphalus flabellatus* |  | 0 | 0 | 20 | 0 |
| *Asteromphalus* spp. |  | 0 | 0 | 0 | 40 |
| *Bacillaria paxillifera* | Bpax | 382 | 460 | 440 | 480 |
| *Bacteriastrum delicatulum* |  | 0 | 0 | 0 | 160 |
| *Bacteriastrum hyalinum* |  | 0 | 0 | 0 | 80 |
| *Bacteriastrum* spp. |  | 0 | 0 | 0 | 320 |
| *Bellerochea malleus* |  | 0 | 0 | 64 | 1000 |
| cf. *Campyloneis* spp. |  | 0 | 0 | 20 | 0 |
| Centric group (<10 µm Ø) | Cen <10 | 700 | 380 | 120 | 80 |
| Centric group (10-20 µm Ø) | Cen 10-20 | 3120 | 5400 | 43240 | 1160 |
| Centric group (20-40 µm Ø) | Cen 20-40 | 240 | 400 | 1080 | 2040 |
| Centric group (>40 µm Ø) | Cen >40 | 0 | 80 | 40 | 120 |
| *Cerataulina pelagica* | Cpel | 213 | 0 | 200 | 80 |
| *Chaetoceros* cf. *affinis* | CHaff | 0 | 20 | 2800 | 4077 |
| *Chaetoceros curvisetus* | CHcur | 0 | 213 | 2680 | 55037 |
| *Chaetoceros* cf. *danicus* |  | 0 | 0 | 20 | 1600 |
| *Chaetoceros* cf. *decipiens* |  | 0 | 0 | 0 | 1019 |
| *Chaetoceros densus* |  | 0 | 0 | 1160 | 16840 |
| *Chaetoceros* cf. *lorenzianus* |  | 0 | 0 | 20 | 40 |
| *Chaetoceros* cf. *rostratus* |  | 0 | 0 | 0 | 120 |
| *Chaetoceros* cf. *similis* |  | 40 | 0 | 0 | 0 |
| *Chaetoceros* cf. *simplex* |  | 0 | 0 | 120 | 0 |
| *Chaetoceros socialis* |  | 0 | 0 | 0 | 360 |
| *Chaetoceros* spp. | CHA | 145 | 1320 | 680 | 78478 |
| *Climaconeis inflexa* |  | 0 | 3080 | 0 | 0 |
| *Cocconeis* sp. |  | 40 | 0 | 20 | 0 |
| *Corethron hystrix* | Chys | 0 | 0 | 40 | 80 |
| *Coscinodiscus* spp. | COS | 4240 | 2640 | 800 | 160 |
| *Coscinodiscus wailesii* |  | 0 | 0 | 40 | 0 |
| *Cylindrotheca closterium* | Cclos | 84560 | 1440 | 48800 | 23760 |
| *Cymatosira* spp. |  | 24 | 0 | 0 | 0 |
| *Dactyliosolen blavyanus/Guinardia flaccida* | Dbla/Gfla | 0 | 40 | 720 | 820 |
| *Dactyliosolen fragilissimus* | Dfra | 50 | 720 | 720 | 1200 |
| *Dactyliosolen phuketensis* | Dphu | 0 | 720 | 2240 | 6200 |
| *Dactyliosolen* spp. |  | 0 | 0 | 20 | 240 |
| cf. *Delphineis surirella* | Dsur | 0 | 0 | 160 | 6000 |
| *Detonula pumila* | Dpum | 0 | 160 | 480 | 680 |
| *Diploneis* cf. *bombus* | Dbom | 760 | 80 | 60 | 60 |
| *Diploneis* cf. *didyma* | Ddid | 50 | 20 | 20 | 40 |
| *Diploneis* cf. *splendida* |  | 156 | 0 | 0 | 0 |
| *Diploneis* spp. | DIP | 50 | 80 | 20 | 20 |
| *Ditylum brightwellii* | Dbri | 160 | 320 | 560 | 400 |
| *Entomoneis* spp. |  | 0 | 20 | 80 | 0 |
| *Eucampia zoodiacus* | Ezoo | 0 | 160 | 240 | 7840 |
| *Eupyxidicula turris* |  | 0 | 0 | 0 | 520 |
| cf. *Fragilaria* spp. |  | 0 | 160 | 0 | 0 |
| cf. *Gomphonema* spp. | GOM | 1867 | 160 | 0 | 1560 |
| *Grammatophora oceanica* | Goce | 160 | 320 | 140 | 40 |
| *Grammatophora* spp. | GRA | 55 | 120 | 96 | 0 |
| *Guinardia delicatula* | Gdel | 4960 | 5600 | 14480 | 23600 |
| *Guinardia striata* | Gstr | 240 | 5040 | 19680 | 5600 |
| *Gyrosigma fasciola* | Gfas | 50 | 80 | 0 | 0 |
| *Gyrosigma* spp./*Pleurosigma* spp. | GYR/PLE | 2067 | 2540 | 3600 | 40 |
| *Helicotheca tamesis* | Htam | 767 | 533 | 96 | 160 |
| *Hemiaulus hauckii* | Hhau | 0 | 0 | 80 | 1240 |
| *Lauderia annulata* |  | 0 | 0 | 0 | 160 |
| *Leptocylindrus* cf. *danicus* | Ldan | 1080 | 17840 | 8640 | 71680 |
| *Leptocylindrus* cf. *minimus* | Lmin | 18 | 0 | 40 | 16400 |
| *Licmophora* spp. | LIC | 280 | 80 | 40 | 240 |
| *Melosira nummuloides* | Mnum | 800 | 160 | 80 | 40 |
| *Meuniera membranacea* | Mmem | 0 | 80 | 20 | 2040 |
| *Navicula* cf. *longissima* |  | 0 | 0 | 0 | 40 |
| *Navicula* spp. | NAV | 800 | 400 | 280 | 920 |
| *Neocalyptrella robusta* |  | 0 | 0 | 0 | 20 |
| *Nitzschia* cf. *sigma* | Nsig | 1147 | 500 | 400 | 0 |
| *Nitzschia* spp. | NIT | 10880 | 960 | 6560 | 48560 |
| *Paralia sulcata* | Psul | 0 | 40 | 0 | 240 |
| Pennate group (<10 µm) | Pen <10 | 400 | 20 | 120 | 120 |
| Pennate group (10-40 µm) | Pen 10-40 | 6880 | 65160 | 6640 | 1000 |
| Pennate group (40-80 µm) | Pen 40-80 | 320 | 2160 | 200 | 760 |
| Pennate group (> 80 µm) | Pen >80 | 550 | 840 | 560 | 160 |
| *Podosira stelligera* |  | 53 | 0 | 80 | 0 |
| *Proboscia alata* | Pala | 67 | 0 | 40 | 28280 |
| *Proboscia indica* | Pind | 0 | 0 | 0 | 120 |
| cf. *Pseudofalcula hyalina* |  | 25920 | 0 | 0 | 0 |
| *Pseudo-nitzschia delicatissima*-group* | PSEdel | 36 | 720 | 400 | 1920 |
| *Pseudo-nitzschia seriata*-group* | PSEser | 0 | 400 | 120 | 25320 |
| *Rhizosolenia* cf. *imbricata* | Rimb | 1956 | 7360 | 25600 | 3840 |
| *Rhizosolenia* cf. *setigera* | Rset | 22 | 80 | 320 | 160 |
| *Rhizosolenia* spp. | RHI | 0 | 0 | 40 | 40 |
| *Rhizosolenia* cf. *styliformis* | Rsty | 0 | 0 | 80 | 40 |
| cf. *Scoliotropis* spp. | SCO | 300 | 587 | 320 | 80 |
| *Skeletonema marinoi* | Smar | 1120 | 2000 | 5680 | 1600 |
| *Striatella unipunctata* |  | 0 | 0 | 120 | 20 |
| *Surirella* spp. | SUR | 560 | 20 | 40 | 20 |
| *Thalassionema nitzschioides* | Tnit | 54240 | 4640 | 10200 | 3640 |
| *Thalassiosira* spp. (>10 µm) | THA >10 | 400 | 320 | 200 | 40 |
| *Thalassiosira* spp. (<10 µm) | THA <10 | 4480 | 6360 | 360 | 160 |
| *Thalassiosira subtilis* | Tsub | 94 | 240 | 7200 | 1480 |
| *Thalassiothrix longissima* | Tlon | 0 | 0 | 0 | 230 |
| *Trachyneis* spp. |  | 27 | 0 | 0 | 0 |
| *Triceratium* spp. |  | 18 | 0 | 0 | 0 |
| *Trieres mobiliensis* | Tmob | 100 | 400 | 240 | 60 |
| *Trieres sinensis* |  | 0 | 0 | 40 | 0 |
| *Zygoceros rhombus* |  | 0 | 0 | 80 | 0 |
| **DINOPHYCEAE** |  |  |  |  |  |
| *Achradina pulchra* |  | 0 | 0 | 0 | 40 |
| *Alexandrium* spp.* | ALE | 160 | 100 | 200 | 200 |
| *Amphidinium* cf. *crassum* |  | 0 | 0 | 0 | 20 |
| *Amphidinium* cf. *operculatum** |  | 0 | 0 | 0 | 40 |
| *Amphidinium* cf. *sphenoides* |  | 0 | 80 | 0 | 40 |
| *Amphidinium* spp. | AMP | 40 | 80 | 80 | 1000 |
| *Azadinium caudatum* var. *caudatum* |  | 0 | 0 | 0 | 16 |
| *Azadinium caudatum* var. *margalefii* |  | 0 | 0 | 0 | 16 |
| *Cochlodinium* spp. | COC | 80 | 160 | 160 | 160 |
| *Cucumeridinium coeruleum* |  | 0 | 0 | 0 | 40 |
| *Cucumeridinium* spp. |  | 0 | 0 | 0 | 20 |
| *Dinophysis acuminata** | Dacum | 0 | 0 | 0 | 160 |
| *Dinophysis acuta** | Dacu | 0 | 0 | 0 | 160 |
| *Dinophysis caudata** | Dcau | 0 | 0 | 64 | 260 |
| *Dinophysis fortii** |  | 0 | 0 | 0 | 20 |
| *Dinophysis ovum** | Dovu | 0 | 0 | 20 | 880 |
| *Diplopsalis* group | DIPLOP | 50 | 80 | 300 | 160 |
| cf. *Erythropsidinium* spp. |  | 0 | 0 | 0 | 80 |
| cf. *Fragilidium* spp. |  | 0 | 0 | 160 | 48 |
| *Gonyaulax* cf. *digitale* |  | 0 | 0 | 0 | 160 |
| *Gymnodinium catenatum** | Gcat | 0 | 0 | 1184 | 4720 |
| *Gymnodinum* spp. | GYM | 200 | 160 | 240 | 60381 |
| *Gyrodinium* cf. *spirale* | Gspi | 0 | 0 | 80 | 240 |
| *Gyrodinium fusiforme* | Gfus | 0 | 0 | 0 | 180 |
| *Gyrodinium* spp. | GYR | 50 | 53 | 240 | 560 |
| *Heterocapsa* spp./*Azadinium* spp.* | HET/AZA | 94 | 80 | 240 | 360 |
| *Histioneis* cf. *marchesonii* |  | 0 | 0 | 0 | 20 |
| *Karenia bicuneiformis** |  | 0 | 0 | 0 | 40 |
| *Karenia mikimotoi** | Kmik | 0 | 0 | 40 | 80 |
| *Karenia selliformis** |  | 0 | 0 | 40 | 0 |
| *Karenia* spp. < 20 µm* | KAR <20 | 80 | 40 | 80 | 120 |
| *Karenia* spp. > 20 µm* | KAR >20 | 0 | 80 | 80 | 40 |
| *Kryptoperidinium triquetrum* |  | 0 | 0 | 20 | 0 |
| *Lebouridinium glaucum* | Lgla | 0 | 80 | 40 | 920 |
| *Lingulodinium polyedra** |  | 0 | 0 | 0 | 480 |
| *Mesoporos perforatus* |  | 0 | 20 | 0 | 0 |
| *Noctiluca scintillans* | Nsci | 0 | 20 | 20 | 80 |
| *Ostreopsis* spp.* |  | 0 | 0 | 0 | 40 |
| *Oxytoxum caudatum* |  | 0 | 0 | 20 | 0 |
| *Oxytoxum* cf. *adriaticum* |  | 0 | 0 | 0 | 40 |
| *Oxytoxum* cf. *turbo* |  | 0 | 0 | 0 | 20 |
| *Oxytoxum laticeps* |  | 0 | 0 | 0 | 80 |
| *Oxytoxum* spp. | OXY | 0 | 13 | 0 | 80 |
| *Oxytoxum variabile* |  | 0 | 0 | 0 | 80 |
| *Phalacroma rotundatum** | Prot | 0 | 0 | 0 | 40 |
| *Podolampas bipes* |  | 0 | 0 | 0 | 280 |
| *Podolampas palmipes* |  | 0 | 20 | 0 | 20 |
| *Polykrikos kofoidii* |  | 40 | 0 | 40 | 0 |
| *Polykrikos schwartzii* |  | 0 | 0 | 0 | 20 |
| *Preperidinium* spp. |  | 0 | 0 | 0 | 20 |
| *Pronoctiluca pelagica* |  | 0 | 0 | 0 | 120 |
| *Prorocentrum* cf. *compressum* |  | 0 | 0 | 0 | 80 |
| *Prorocentrum* cf. *concavum** |  | 0 | 0 | 0 | 20 |
| *Prorocentrum cordatum** | Pcor | 40 | 320 | 920 | 147784 |
| *Prorocentrum lima** |  | 0 | 20 | 0 | 40 |
| *Prorocentrum micans* | Pmic | 120 | 1000 | 120 | 240 |
| *Prorocentrum scutellum* |  | 0 | 40 | 0 | 240 |
| *Prorocentrum* spp. |  | 0 | 20 | 0 | 0 |
| *Prorocentrum triestinum* |  | 80 | 20 | 0 | 0 |
| *Protoceratium reticulatum** |  | 0 | 0 | 0 | 40 |
| *Protoperidinium bipes* | Pbip | 53 | 80 | 400 | 160 |
| *Protoperidinium brevipes* | Pbre | 160 | 120 | 128 | 80 |
| *Protoperidinium* cf. *achromaticum* |  | 200 | 0 | 0 | 0 |
| *Protoperidinium* cf. *cerasus* | Pcer | 0 | 40 | 160 | 240 |
| *Protoperidinium* cf. *crassipes* |  | 0 | 0 | 0 | 80 |
| *Protoperidinium* cf. *curtipes* | Pcur | 0 | 0 | 32 | 320 |
| *Protoperidinium* cf. *curvipes* |  | 0 | 0 | 40 | 60 |
| *Protoperidinium* cf. *depressum* |  | 0 | 0 | 0 | 40 |
| *Protoperidinium* cf. *globulus* |  | 40 | 0 | 0 | 0 |
| *Protoperidinium* cf. *oblongum* |  | 0 | 0 | 0 | 40 |
| *Protoperidinium* cf. *ovatum* |  | 0 | 0 | 240 | 40 |
| *Protoperidinium* cf. *oviforme* |  | 0 | 0 | 0 | 80 |
| *Protoperidinium* cf. *ovum* |  | 0 | 0 | 0 | 40 |
| *Protoperidinium* cf. *pyriforme* |  | 0 | 0 | 0 | 40 |
| *Protoperidinium* cf. *subinerme* | Psub | 53 | 120 | 80 | 40 |
| *Protoperidinium* cf. *thulesense* |  | 0 | 20 | 0 | 0 |
| *Protoperidinium diabolus* | Pdia | 0 | 0 | 40 | 720 |
| *Protoperidinium divergens* |  | 0 | 0 | 20 | 40 |
| *Protoperidinium quinquecorne* |  | 0 | 160 | 0 | 0 |
| *Protoperidinium* spp. | PROT | 160 | 160 | 240 | 320 |
| *Protoperidinium steinii* |  | 0 | 0 | 0 | 40 |
| *Pseliodinium vaubanii* |  | 0 | 0 | 0 | 100 |
| *Scrippsiella* spp. group | SCR gr | 480 | 2160 | 5360 | 16-3240 |
| *Spatulodinium pseudonoctiluca* |  | 0 | 0 | 0 | 40 |
| *Torodinium robustum* | Trob | 0 | 40 | 120 | 400 |
| *Torodinium teredo* |  | 0 | 0 | 0 | 16 |
| *Torquentidium* cf. *helix* |  | 0 | 0 | 0 | 20 |
| *Triadinium polyedricum* |  | 0 | 0 | 0 | 80 |
| *Tripos candelabrum* |  | 0 | 0 | 0 | 40 |
| *Tripos* cf. *massiliensis* |  | 0 | 0 | 0 | 40 |
| *Tripos furca* | Tfur | 18 | 27 | 80 | 1280 |
| *Tripos fusus* | Tfus | 0 | 20 | 20 | 912 |
| *Tripos horridus* | Thor | 0 | 0 | 0 | 40 |
| *Tripos lineatus* | Tlin | 0 | 0 | 0 | 560 |
| *Tripos muelleri* | Tmue | 0 | 0 | 0 | 240 |
| *Tripos* spp. |  | 0 | 0 | 0 | 40 |
| *Tripos trichoceros* |  | 0 | 0 | 0 | 20 |
| Athecate dinoflagellates group (> 15 µm) | Athecate gr | 300 | 680 | 740 | 41680 |
| Small dinoflagellates group (< 15 µm) | Small din | 1800 | 920 | 920 | 42280 |
| **CRYPTOPHYCEAE** |  |  |  |  |  |
| Cryptophytes | CRY | 370096 | 177341 | 68494 | 35878 |
| **EUGLENOPHYCEAE** |  |  |  |  |  |
| Euglenophytes | EUG | 20040 | 8240 | 4080 | 1640 |
| **PRASINOPHYCEAE** |  |  |  |  |  |
| Prasinophytes | PRA | 18346 | 189571 | 0 | 1631 |
| *Pterosperma cristatum* |  | 0 | 60 | 0 | 0 |
| *Pterosperma moebii* |  | 0 | 0 | 0 | 40 |
| *Pterosperma* spp. |  | 0 | 0 | 20 | 40 |
| **CHLOROPHYCEAE** |  |  |  |  |  |
| *Ankistrodesmus* cf. *arcuatus* | Aarc | 80 | 40 | 20 | 0 |
| *Monoraphidium* cf. *caribeum* |  | 36 | 0 | 0 | 0 |
| *Monoraphidium contortum* |  | 0 | 40 | 40 | 0 |
| *Monoraphidium* cf. *flexuosum* |  | 0 | 0 | 40 | 0 |
| *Monoraphidium* cf. *griffithii* | Mgri | 107 | 240 | 160 | 16 |
| *Monoraphidium* spp. | MON | 40 | 0 | 40 | 40 |
| *Scenedesmus* spp. |  | 160 | 80 | 80 | 0 |
| **CRYSOPHYCEAE** |  |  |  |  |  |
| *Dictyocha fibula* | Dfib | 0 | 0 | 20 | 440 |
| *Octactis octonaria* | Ooct | 0 | 40 | 64 | 40 |
| *Octactis speculum* | Ospe | 0 | 0 | 60 | 100 |
| **CYANOPHYCEAE** |  |  |  |  |  |
| *Merismopedia* cf. *elegans* | Mele | 0 | 0 | 0 | 5440 |
| *Merismopedia* cf. *hyalina* |  | 0 | 0 | 0 | 640 |
| *Merismopedia* spp. |  | 600 | 0 | 0 | 0 |
| *Oscillatoria* spp. |  | 0 | 5860 | 0 | 0 |
| cf. *Pseudanabaena* spp. |  | 0 | 0 | 960 | 0 |
| **PRYMNESIOPHYCEAE** |  |  |  |  |  |
| *Phaeocystis* spp.* | PHA | 0 | 0 | 0 | 23960 |
| **OTHER SMALL FLAGELLATES** |  |  |  |  |  |
| Other small flagellates (< 15 µm) | Small flag | 153295 | 25480 | 30985 | 4892 |
| **TOTAL TAXONOMIC ENTITIES** |  | **87** | **103** | **133** | **179** |

Table S3 **–** Summary of the similarity percentages routine (SIMPER) analysis between the factor Year. The average abundances (log X+1 transformed), respective contributions (“Contrib. (%)”), and the cumulative contributions (“Cumul (%)”) of each taxonomic entity to the observed dissimilarity between 2018 and 2019 are provided. Only the taxonomic entities that explained 30% of the cumulative dissimilarity are shown.

| **SIMPER average dissimilarity = 51.70** | | |  |  |
| --- | --- | --- | --- | --- |
| **Taxonomic entities** | **Average Abundance (log X+1)** | | **Contrib. (%)** | **Cumul. (%)** |
|  | **2018** | **2019** |  |  |
| Small flag | 4.97 | 7.38 | 2.58 | 2.58 |
| Gdel | 3.09 | 4.06 | 2.22 | 4.80 |
| THA <10 | 2.12 | 3.87 | 2.17 | 6.97 |
| Smar | 2.70 | 3.75 | 2.10 | 9.07 |
| Tnit | 4.92 | 4.67 | 1.92 | 10.99 |
| CHA | 3.10 | 2.24 | 1.89 | 12.89 |
| Ldan | 2.76 | 2.26 | 1.87 | 14.76 |
| SCR gr | 3.59 | 3.90 | 1.79 | 16.55 |
| GYR/PLE | 3.45 | 3.57 | 1.78 | 18.34 |
| Nsig | 2.63 | 2.97 | 1.66 | 20.00 |
| COS | 4.44 | 3.30 | 1.61 | 21.61 |
| Athecate gr | 4.33 | 3.79 | 1.53 | 23.14 |
| Pen 40-80 | 2.60 | 3.15 | 1.51 | 24.65 |
| NIT | 5.36 | 4.61 | 1.50 | 26.15 |
| Cen 20-40 | 3.53 | 3.62 | 1.50 | 27.65 |
| CRY | 9.32 | 8.40 | 1.50 | 29.15 |
| Pen >80 | 1.87 | 3.08 | 1.50 | 30.65 |
